# Drug Screening for Autophagy Inhibitors Based on the Dissociation of Beclin1-Bcl2 Complex Using BiFC Technique and Mechanism of Eugenol on Anti-Influenza A Virus Activity

**DOI:** 10.1371/journal.pone.0061026

**Published:** 2013-04-16

**Authors:** Jian-Ping Dai, Xiang-Feng Zhao, Jun Zeng, Qian-Ying Wan, Jia-Cai Yang, Wei-Zhong Li, Xiao-Xuan Chen, Ge-Fei Wang, Kang-Sheng Li

**Affiliations:** 1 Department of Microbiology and Immunology, Shantou University Medical College, Shantou, Guangdong, People’s Republic of China; 2 Department of Veterinary Medicine, University of Maryland, College Park, Maryland, United States of America; National Institute for Viral Disease Control and Prevention, CDC, China

## Abstract

Autophagy is involved in many human diseases, such as cancer, cardiovascular disease and virus infection, including human immunodeficiency virus (HIV), hepatitis C virus (HCV), influenza A virus (IAV) and coxsackievirus B3/B4 (CVB3/B4), so a drug screening model targeting autophagy may be very useful for the therapy of these diseases. In our study, we established a drug screening model based on the inhibition of the dissociation of Beclin1-Bcl2 heterodimer, an important negative regulator of autophagy, using bimolecular fluorescence complementation (BiFC) technique for developing novel autophagy inhibitors and anti-IAV agents. From 86 examples of traditional Chinese medicines, we found *Syzygium aromaticum* L. had the best activity. We then determined the anti-autophagy and anti-IAV activity of eugenol, the major active compound of *Syzygium aromaticum* L., and explored its mechanism of action. Eugenol could inhibit autophagy and IAV replication, inhibited the activation of ERK, p38MAPK and IKK/NF-κB signal pathways and antagonized the effects of the activators of these pathways. Eugenol also ameliorated the oxidative stress and inhibited the expressions of autophagic genes. We speculated that the mechanism underlying might be that eugenol inhibited the oxidative stress and the activation of ERK1/2, p38MAPK and IKK/NF-κB pathways, subsequently inhibited the dissociation of Beclin1-Bcl2 heterodimer and autophagy, and finally impaired IAV replication. These results might conversely display the reasonableness of the design of our screening model. In conclusion, we have established a drug screening model for developing novel autophagy inhibitor, and find eugenol as a promising inhibitor for autophagy and IAV infection.

## Introduction

Influenza A virus (IAV) is a severe risk for the public health. The drugs-resistant IAV mutants to the current anti-IAV drugs have been reported frequently [1], [2], [3], so developing novel anti-IAV drugs is still urgent. It is well known that IAV infection can induce autophagy. Gannage M. et al have shown that IAV M2 can block autophagosome fusion with lysosomes [4]. Gregoire I.P. et al have analyzed the interactions between 9 IAV proteins and 44 human autophagy-associated proteins using yeast two-hybrid technique and shown that IAV NP protein can interact with ATG4C, BNIP3 and GOPC proteins, NS1 can interact with ATG5 and GOPC, NS2 can interact with ATG5, ATG9A IRGM and UVRAG, PB1-F2 can interact with ATG5 and IRGM, PB2 can interact with SQSTM1, and M2 can interact with BECN1 [5]. It is also reported that autophagy is involved in IAV replication [6], though some researchers have not detected the significant decrease of IAV titer upon autophagy inhibition [4], [5], [7], Zhou Z. et al have reported that pretreatment or treatment of MDCK or A549 cells with 3-MA or wortmannin, or depletion of LC3 and Beclin 1 by siRNA technique, greatly reduce the yield of extracellular and intracellular virus and impair the accumulation of IAV M1 and M2 proteins [6]. Sun Y. et al have shown that H5N1 induces autophagic cell death in alveolar epithelial cells through a pathway involving Akt/TSC2/mTOR, When treatment with the autophagy inhibitor 3-MA prophylacticly and therapeuticly, or knockdown of the autophagic genes Atg5 and Beclin1, substantially inhibit H5N1-induced autophagic cell death and ameliorate the acute lung injury and mortality [7]. They suggest that autophagy-blocking agents may be useful as prophylactics and therapeutics against H5N1 infection [7]. So autophagy inhibition is now thought to be a possible and novel strategy for developing novel anti-IAV drugs [6], [7], [8].

Regulation of macroautophagy (hereafter referred to as autophagy) is complicated (**Figure S1**). Upstream of mTOR, the TSC1/2 complex accepts the regulations of several signal pathways, such as PI3KCI/Akt, LKB1/AMPK, MEM/ERK and HIF-1/REDD1, and negatively regulates mTOR activity through directly stimulating GTP hydrolysis of Rheb [9]. Downstream of mTOR, there is a very important regulator Beclin1, which has been proved as a major target for manipulation of autophagy by many viruses, such as human immunodeficiency virus (HIV), hepatitis C virus (HCV), herpes simplex virus (HSV) and Coxsackievirus B3/4 [10]. It participates both in the biogenesis and degradation of autophagosomes via its interaction with different complexes. Beclin1 exists in three complexes: Atg14L complex (Atg14L, Beclin 1, Vps34 and p150), UVRAG complex (UVRAG, Beclin 1, Vps34 and p150) and Rubicon complex (Rubicon, UVRAG, Beclin 1, Vps34 and p150) [10]. There is a dynamic exchange between these Beclin1 complexes [11]. In addition, there are many other proteins, such as Bif-1, Ambra1, nPIST, VMP1, SLAM, PINK1 and Survivin, interacting with Beclin1 [12]. In a word, Beclin1 binds with Vps34 and p150 to form a core complex, and further interacts with other proteins to form different complexes to play several important roles in autophagy regulation.

**Figure 1:**
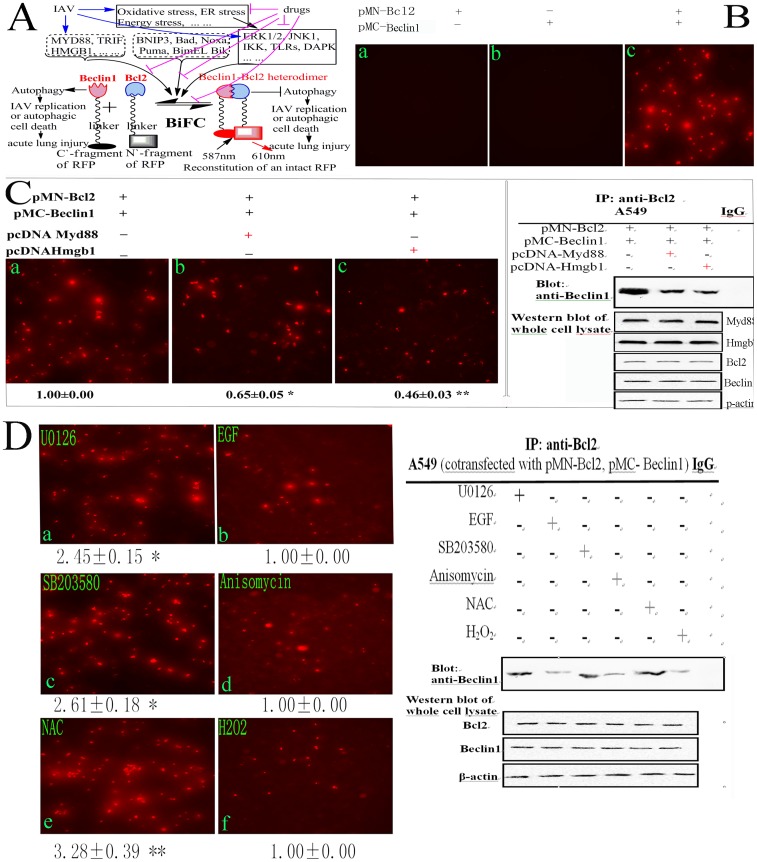
Establishment of our drug screening model. (A) Schematic representation of the drug screening model. Beclin1 plays several important roles in autophagy, the dissociation of Beclin1 from Bcl2 is essential for autophagy. Autophagy either supports IAV replication or induces autophagic cell death which may result in acute lung injury (left). Bcl2 binds with Beclin1 to form Beclin1-Bcl2 heterodimer and inhibits autophagy (right). Beclin1-binding proteins (such as MyD88, TRIF and HMGB1), Bcl2-binding proteins (such as BNIP3, Bad, Noxa, Puma, BimEL and Bik) and the activations of ERK1/2, JNK1, IKK, TLRs and DAPK signal pathways under oxidative stress, endoplasmic reticulum (ER) stress and energy stress can promote the dissociation of Beclin1 from Bcl2, and finally promote autophagy. IAV infection can elevate the expressions of many aforementioned proteins and activate aforementioned signal pathways. Drugs inhibiting oxidative stress, ER stress, energy stress, and the activations of ERK1/2, JNK1, IKK, TLRs and DAPK signal pathways may inhibit the dissociation of Beclin1 from Bcl2, sequentially inhibit autophagy, and finally impair IAV replication or inhibit autophagic cell death and acute lung injury. In our study, Beclin1 and Bcl2 were fused with C’- and N’- fragments of a red fluorescence protein (RFP), respectively. The amino acid sequence of the linker was RPACKIPNDLKQKVMNH. After cotransfection, the intact RFP would reconstitute through the interaction of Beclin1 and Bcl2. The fluorescence intensity (FI) of the reconstituted RFP, which was determined at 610 nm after excitation at 587 nm using a microplate reader (Tecan infinite M1000), represents the level of the Beclin1-Bcl2 heterodimer, which is positively relevant with the degree of autophagy inhibition and antiviral activity. (B) Reconstitution of RFP. A549 cells were transfected or cotransfected with pMN-Bcl2 and pMC-Beclin1, after 8 h, only cotransfected cells appeared a lot of red fluorescence. (C) The influence of HMGB1 and MyD88 on the Beclin1-Bcl2 heterodimer. Beclin1-binding proteins HMGB1 and MyD88 were expected to disrupt the Beclin1-Bcl2 heterodimer, after cotransfection as the graph indicated, the FI was really significantly decreased, and the cells of same batch were subjected to the co-immunoprecipitation (co-IP) assay (right), the results satisfied our expectation. (D) The influence of ERK1/2 inhibitor (U0126, 10 µM), ERK1/2 activator (EGF, 100 ng/ml), JNK/p38 inhibitor (SB203580, 40 µM), p38 MAPK activator (anisomycin, 10 µM), antioxidant (NAC, 2 mM) and oxidant (H2O2, 100 µM) on the dissociation of Beclin1-Bcl2 heterodimer. After cotransfection, A549 cells were treated with these inhibitors and activators, after 8 h, the FI was measured, the inhibitors (U0126, SB203580) and antioxidant (NAC) could significantly elevate the FI, which meant that they could inhibit the dissociation of Beclin1 from Bcl2, as comparing with their corresponding activators (EGF, anisomycin) and oxidant (H_2_O_2_), the cells of the same batch were also subjected to the co-IP assay (right), the results also satisfied our expectation. Normal rabbit IgG was used as a control in co-IP assay. Data shown were the mean ± SD of three independent experiments and shown as the fold change to the corresponding control. *P<0.05, **P<0.01.

Among Beclin1-binding proteins, Bcl2 is an important inhibitor for autophagy, the dissociation of Beclin1 from Bcl2 is essential for autophagy and is regulated by many proteins and signal pathways (**Figure 1(A)** and **Figure S1**): **(1)** the competitive displacement of Beclin1 by Bcl2-binding proteins, such as BNIP3, Bad, Noxa, Puma, BimEL and Bik [13]; **(2)** the competitive displacement of Bcl-2 by Beclin1-binding proteins, such as MyD88, TRIF and HMGB1 [14]; **(3)** ERK1/2- or JNK1-mediated phosphorylation of Bcl2 or DAPK-mediated phosphorylation of Beclin1 promote the dissociation of Beclin1-Bcl2 heterodimer and further enhance autophagy, JNK1, ERK and DAPK signals are further regulated by oxidative stress, energy stress and endoplasmic reticulum (ER) stress [12], [15]; **(4)** the TRAF6-mediated ubiquitinating and A20-mediated deubiquitinating of Beclin1 regulate the dissociation of Beclin1-Bcl2 heterodimer [12], [14], and the TLRs signaling enhances the interaction of MyD88 and TRIF with Beclin1, reduces the binding of Beclin1 to Bcl2 and promotes autophagy [14]; **(5)** TAB2/3 can bind with Beclin1, upon induction, TAB2/3 dissociates from Beclin1 and activates IKK, IKK activation is necessary for autophagy [16], [17]. Because Beclin1 plays important roles in autophagy regulation, and Bcl2 is an important inhibitor of Beclin1, so the inhibition of the dissociation of Beclin1-Bcl2 heterodimer is a good target for developing autophagy inhibitor.

IAV influences autophagy not only by its M2 protein binding with Beclin1 [4], but also by increasing the autophagic flux [6], [18]. Our previous research also has shown that IAV can increase the expression of autophagic genes and autophagic flux [19]. Additionally, as indicated in **Figure S1**, IAV infection can activate the IKK, PKC, JNK1, ERK and TLRs/MyD88/TRIF/TRAF6 signal pathways, all of which can lead to the elevation of autophagy. IAV infection also can result in oxidative stress by Nox2 NADPH oxidase and produces a lot of reactive oxygen species (ROS). H_2_O_2_ can promote autophagy by inhibiting Atg4 [20]. ROS can induce autophagic cell death through FoxO1 and Atg7 [21]. Recently, Sun Y. et al have reported that autophagic cell death is responsible for the acute lung injury and the high mortality rate (60%) induced by IAV H5N1 and IAV hemagglutinin (HA) can stimulate autophagic flux [7]. Ma J. et al have shown that IAV H5N1 causes autophagic cell death through TSC1/2-mTOR signaling [8]. Both of them have shown that inhibition of autophagic flux significantly reduces the H5N1-mediated cell death and mortality of mice [7], [8]. How can we control the autophagic flux induced by IAV? As aforementioned, we select the inhibition of the dissociation of Beclin1-Bcl2 heterodimer as our target to control autophagic flux. Based on the dissociation of Beclin1-Bcl2 heterodimer, we have established a drug screening model using bimolecular fluorescence complementation (BiFC) technique (**Figure 1** **A)**. BiFC technique is based on the principle that two non-fluorescent fragments of a fluorescent protein are brought together by the interaction of proteins fused to each fragment and reconstructs an intact fluorescence protein, then quantitating the fluorescence intensity (FI) of the reconstructed fluorescence protein to display the influence of drug on the interaction of interest proteins in living cells [22], [23], [24].

Our purpose was first to establish a drug screening model to find out novel autophagy inhibitors, and then detect the anti-IAV activity of these autophagy inhibitors. Using this model, we screened 86 examples of traditional medicinal plants, and found *Syzygium aromaticum* L. had the best activity, next we detected whether eugenol, the major active compound of *Syzygium aromaticum* L., had anti-IAV activity, then explored the mechanism of action, such as antioxidation, the influences on the ERK/JNK/p38 MAPK and IKK/NF-κB pathways, and the expressions of autophagic genes, all of which were important regulators of the dissociation of Beclin1-Bcl2 heterodimer as above mentioned, and thus conversely displayed the reasonableness of the design of our drug screening model.

## Results

### Establishment of Our Drug Screening Model and the Result of Drug Screening Assay

**Figure 2:**
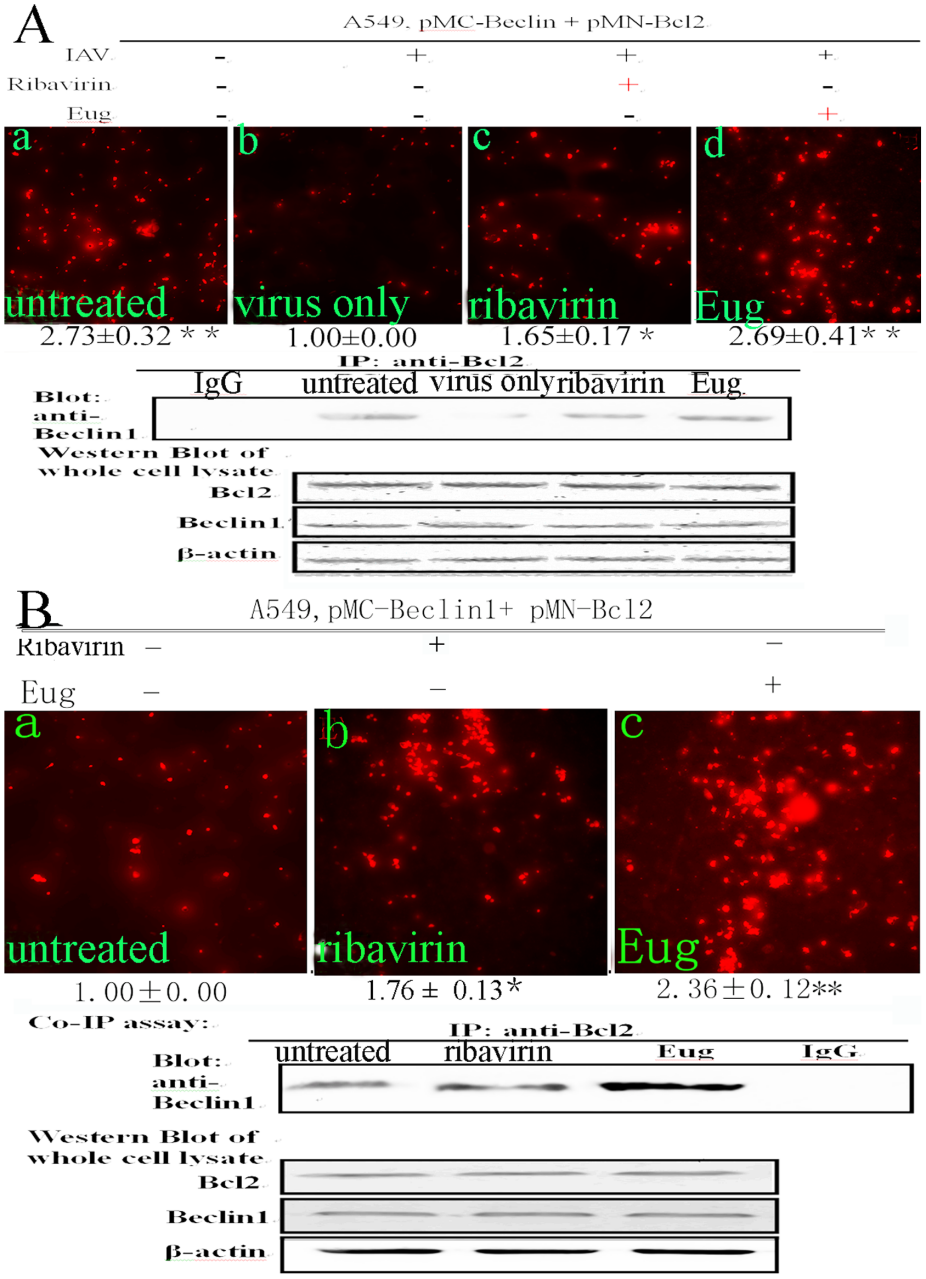
The influences of eugenol on the Beclin1-Bcl2 heterodimer with and without IAV infection. (A) After IAV infection, eugenol could inhibit the dissociation of Beclin1 from Bcl2. After cotransfection with pMC-Beclin1 and pMN-Bcl2, A549 cells were infected with IAV (A/ShanTou/169/06(H1N1)) (MOI = 2.0) and treated with ribavirin (25 µg/ml) and eugenol (5 µg/mL, before this test we had determined the cytotoxicity of eugenol, as shown in Figure 4(A), the maximal concentration without cytotoxicity was 5 µg/mL, so we chose 5 µg/mL as our test concentration), after 8 h, the FI was measured, and the cells of the same batch were subjected to the co-IP assay (below). (B) Without IAV infection, eugenol also could inhibit the dissociation of Beclin1 from Bcl2. After cotransfection with pMC-Beclin1 and pMN-Bcl2, A549 cells were not infected with IAV but directly treated with ribavirin (25 µg/ml) and eugenol (5 µg/mL), after 8 h, the FI was measured, and the cells of the same batch were subjected to the co-IP assay (below). Data shown were the mean ± SD of three independent experiments and shown as the fold change to the corresponding control. *P<0.05, **P<0.01.

BiFC is a recently emerged technique to study protein–protein interaction in living cells, which allows the direct real time visualization of the protein complex under physiological conditions. In this study, we first constructed two plasmids containing the amino acids 1 to 159 and 160 to 262 of a red fluorescent protein (RFP), respectively, we then inserted human Beclin1 and Bcl2 genes in these two plasmids, respectively. (**Figure 1(A)** **)** The amino acid sequence of the linker was RPACKIPNDLKQKVMNH. After transfection with pMN-Bcl2 or pMC-Beclin1 alone, no red fluorescence could be seen because only N`- or C`-fragment of RFP could not emit red fluorescence. After cotransfection with pMN-Bcl2 and pMC-Beclin1, the intact RFP could reconstruct through the conjugation of Beclin1 and Bcl2 (**Figure 1(B)**). Larger graphs and the ratios of RFP-positive cells could be seen in **Figure S2 A**. As aforementioned, the dissociation of Beclin1-Bcl2 heterodimer could be regulated by Beclin1- and Bcl2- binding proteins, and by ERK1/2, JNK1, p38MAPK and IKK/NF-κB signal pathways, here we designed several experiments to verify whether our screening model could be regulated by these factors. Beclin1-binding proteins MyD88 and HMGB1 were reported to disrupt the Beclin1-Bcl2 heterodimer and promoted autophagy [14], [25], we constructed two eukaryotic expression plasmids pcDNA-MyD88 and pcDNA-HMGB1, and after cotransfection, we found both of them could significantly promote the dissociation of Beclin1-Bcl2 complex, and the fluorescence intensities were significantly decreased, the co-IP assay showed the same results (**Figure 1(C)**). Larger graphs and the ratios of RFP-positive cells could be seen in **Figure S2 B**. Further, we detected if the signal pathways, such as ERK1/2, JNK1 and p38 MAPK, and extracellular stimulus, such as H_2_O_2_ and NAC (N-Acetyl-Cysteine), could influence the dissociation of Beclin1-Bcl2 heterodimer. As shown in **Figure 1(D)**, U0126 (ERK inhibitor, 10 µM), SB203580 (p38 MAPK inhibitor, 40 µM) and NAC (antioxidant, 2 mM) could increase the fluorescence intensity, which meant that they could inhibit the dissociation of Beclin1-Bcl2 heterodimer, as comparing with their corresponding activators EGF (ERK activator, 100 ng/mL), anisomycin (JNK/p38 activator, 10 µM) and H_2_O_2_ (oxidant, 100 µM), the co-IP assay also showed the same result. Larger graphs and the ratios of RFP-positive cells could be seen in **Figure S3**. Moreover, IAV infection could significantly decrease the fluorescence intensity, which meant that IAV infection could promote the dissociation of Beclin1-Bcl2 heterodimer, as comparing with the untreated group (**Figure 2(A)**). Larger graphs and the ratios of RFP-positive cells could be seen in **Figure S4**. These experiments showed that our drug screening model could be sensitively regulated by Beclin1-binding proteins, several signal pathways and IAV infection which satisfied our primary expectation.

Using this screening model, 86 examples of traditional Chinese medicines were investigated. As indicated in **Table S1**, after IAV infection, the FI was significantly decreased, the FI of blank group (BG, untreated group) was 2.78 times to that of the negative group (NC, virus only group). Additionally, there were 15 examples of traditional Chinese medicine could significantly (*P*<0.05) elevate the FI value. Among them, *Syzygium aromaticum* L. had the best activity, and up to now, it had not been reported to possess anti-IAV activity. We then chose it as our drug of interest, and purchased eugenol (purity >98%), the major active compound of *Syzygium aromaticum* L., and further detected its effects of anti-autophagy and anti-IAV activity and explored the mechanism of action. In addition, Z`-factor, a statistical parameter to quantify the suitability of a particular assay for use in a high-throughput screen, of this screening model was 0.5112 (>0.5) which showed that our screening model was valid.

### Eugenol could Inhibit the Dissociation of Beclin1-Bcl2 Heterodimer with or without IAV Infection and the Elevation of Autophagy Induced by IAV

**Figure 3:**
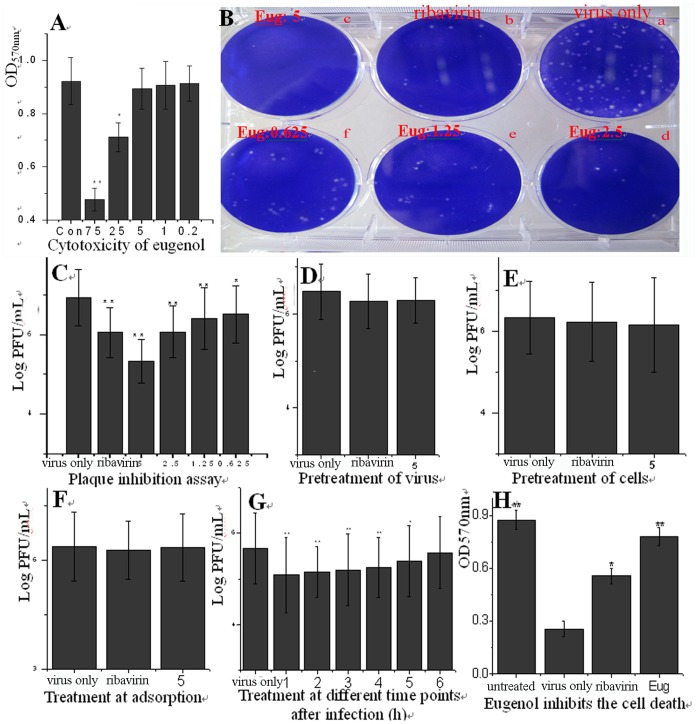
Eugenol inhibited the replication of IAV (A/ShanTou/169/06(H1N1)) and IAV-induced cell death. (A) The cytotoxicity of eugenol determined by MTT method on MDCK cells. Y = −0.006X +0.9125, R^2^ = 0.9798, IC_50_ = 75.28 µg/ml, in the control group (Con), the cells were not treated with any drugs but solvent vehicle (DMSO <0.5%), the maximal concentration without cytotoxicity was 5 µg/mL, and we chose 5 µg/mL as our test concentration,. (B and C) Plaque inhibition assay, at the indicated concentrations, the virus was pretreated with eugenol for 3 h, then infected MDCK cells for 1 h (MOI = 0.001), after washed with PBS 3 times, the cells were cultured in a serial of mediums containing eugenol at the indicated concentrations for 48 h, after centrifuging at 4°C, 8000×g, 10 min, the supernatants were collected and the titers were determined using a plaque assay, the plaques (Ф >1 mm) were counted. Y = −0.3007X +6.8103, R^2^ = 0.9809, EC_50_ = 0.6392 µg/mL. Ribavirin (25 µg/ml) and 0.5%DMSO were used as the positive (ribavirin) and negative (virus only) controls, respectively. (D–G) Time-of-addition assay, (D) Before infection, the virus was pretreated with a medium containing eugenol (5 µg/mL) for 3 h; (E) Before infection, the cells were pretreated with a medium containing eugenol (5 µg/mL) for 3 h; (F) Eugenol (5 µg/mL) was added during the viral adsorption for 1 h and removed by washing 3 times with PBS; (G) Eugenol (5 µg/mL) was added at different time points after virus infection. MOI = 2.0. The incubation time after absorption was 12 h. The other performances were same with the plaque inhibition assay. In the negative control (virus only), the cells were infected with IAV but not treated with any drugs; in the positive control (ribavirin) and eugenol-treated groups, the cells were infected with IAV and treated with ribavirin (25 µg/ml) and eugenol (5 µg/mL), respectively. (H) Eugenol inhibited the cell death induced by IAV infection determined by MTT method. A549 cells were infected with IAV (MOI = 0.01) and treated with Ribavirin (25 µg/ml) and Eugenol (5 µg/mL), after 24 h, the cell viability was determined by MTT method. Data shown were the mean ± SD of three independent experiments performed in triplicate.*P<0.05 and **P<0.001 vs. NC.

In primary screening, the crude extract of *Syzygium aromaticum* L. was used, here we detected the effect of eugenol, the major active compound of *Syzygium aromaticum* L., on the dissociation of Beclin1-Bcl2 heterodimer. Here we chose 5 µg/mL as our test concentration because the maximal concentration of eugenol without cytotoxicity was 5 µg/mL (**Figure 3(A)**). As shown in **Figure 2(A)**, A549 cells were cotransfected with pMN-Bcl2 and pMC- Beclin1, after IAV infection, the FI was significantly decreased (virus only group) comparing with the untreated group. Ribavirin and eugenol could significantly increase the FI comparing with the virus only group, the co-IP assay showed the same results (below). Larger graphs and the ratios of RFP-positive cells could be seen in **Figure S4**. As shown in **Figure 2(B)**, without IAV infection, eugenol also could significantly increase the FI, the co-IP assay showed the same results (below). Larger graphs and the ratios of RFP-positive cells could be seen in **Figure S5**. These experiments showed that eugenol could inhibit the dissociation of Beclin1 from Bcl2 with or without IAV infection.

**Figure 4:**
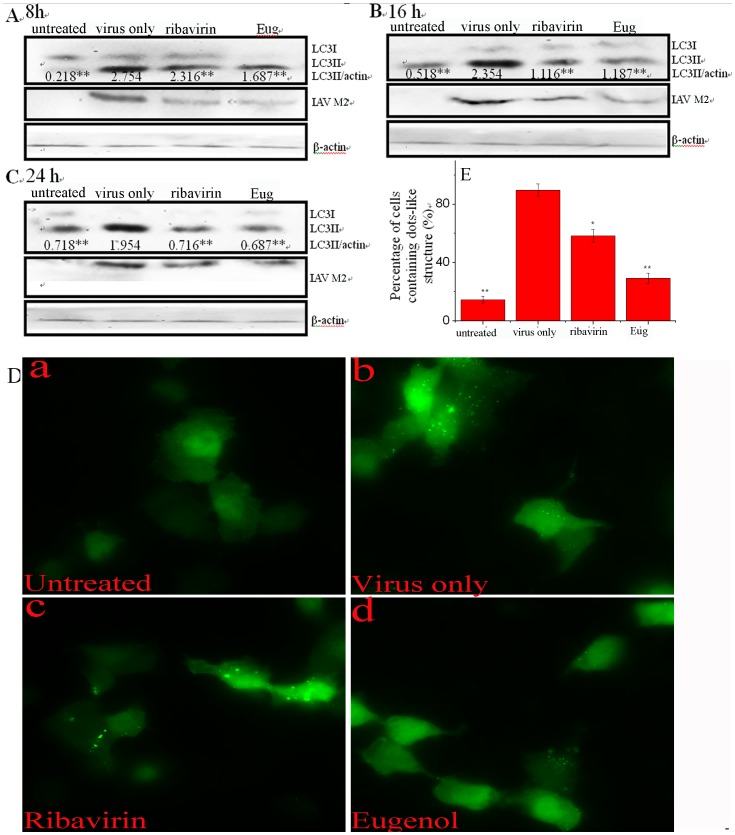
Eugenol inhibited the elevated autophagy after IAV infection (A/ShanTou/169/06 (H1N1)). (A, B and C) Eugenol inhibited the conversion of LC3I to LC3II determined by western blot. In the untreated group, A549 cells were not infected with IAV. In the virus only treated group, A549 cells were infected but not treated with any drugs. In the ribavirin and Eug treated groups, A549 cells were infected and treated with ribavirin (25 µg/ml) and eugenol (5 µg/mL), respectively. MOI = 0.001, the incubation times were 8, 16 and 24 h, respectively. The ratios of LC3-II to β-actin were presented below the blots. At each time point, we also determined the level of IAV M2 protein of each group. (D) Eugenol inhibited the accumulation of autophagosomes determined by EGFP-LC3 assay. In untreated, virus only, ribavirin and Eug groups, A549 cells were transfected with the pEGFP-LC3 plasmid, then performed as above western blot assay, but MOI = 2.0, the incubation time was 8 h. The percentage of cells containing EGFP-LC3 dots to cells expressing EGFP was calculated in 10 fields chosen at random (E). The graphs were obtained from an inverted fluorescence microscope (10×40). Data shown were the mean ± SD of three independent experiments. *P<0.05 and **P<0.01 vs. NC.

As aforementioned, autophagy either supports IAV replication or induces autophagic cell death which may result in acute lung injury [6], [7], [8], here we determined the influence of eugenol on IAV- induced autophagy. We first investigated the influence of eugenol on the transformation of LC3I to LC3II by Western blot. As shown in **Figure 4** **(A, B** and **C),** IAV infection could significantly (*P*<0.01) increase the ratios of LC3-II to β-actin at 8, 16 and 24 h post infection (p.i.). Eugenol (5 µg/mL) could significantly decrease these ratios. Additionally, at each time point, we also determined the levels of IAV M2 protein of each group, which showed when autophagy was inhibited the accumulation of IAV M2 protein was decreased. Next we determined the accumulation of autophagosomes induced by IAV. Here we constructed a plasmid that expressed a fusion protein EGFP-LC3II, which could form dot-like aggregations on autophagosomes. After IAV infection (virus only group), the percentage of cells containing EGFP-LC3 dots to cells expressing EGFP were significantly increased **(** **Figure 4(D** **b)** and **Figure 4(E)** **)**, as compared with the blank group (untreated group). Eugenol (5 µg/mL) could significantly inhibit the dot-like aggregations of EGFP-LC3 **(** **Figure 4(D** **d)** and **Figure 4(E)** **)**, as compared with the virus only group. Larger graphs could be seen in **Figure S6**. These two experiments indicated that eugenol could inhibit the elevation of autophagy induced by IAV.

### Eugenol Inhibited IAV Replication at 1–5 h p.i. and Cell Death Induced by IAV Infection

As aforementioned, the dissociation of Beclin1-Bcl2 heterodimer was crucial for autophagy, the inhibition of the dissociation of Beclin1-Bcl2 heterodimer could inhibit autophagy and autophagy inhibition might inhibit IAV replication [6] or IAV-induced autophagic cell death [7], [8]. Here we determined the anti-IAV activity of eugenol. Before our experiments, we had determined the cytotoxicity of all test drugs. Here we only showed the data of eugenol on MDCK cells. As shown in **Figure 3(A)**, eugenol could significantly inhibit the viability of MDCK cells from 25 to 75 µg/mL, Y = −0.006X +0.9125, R^2^ = 0.9798, IC_50_ = 75.2833 µg/mL. We chose 5 µg/mL as the optimal concentration. We then determined the anti-IAV activity of eugenol using a plaque inhibition assay. As shown in **Figure 3** **(B** and **C)**, eugenol significantly inhibited the replication of IAV in the range of 0.625–5 µg/mL. Y = −0.3007X +6.8103, R^2^ = 0.9809, EC_50_ = 0.6392 µg/mL. Antiviral index (AI) = IC_50_/EC_50_ = 117.78.

In addition, we determined the influence of eugenol on the different stages of IAV life cycle. The results showed that Eugenol could not inactivate IAV directly **(** **Figure 3(D)** **)**, had no influence on cells before infection **(** **Figure 3(E)** **)**, and had no influence on IAV adsorption **(** **Figure 3(F)** **)**. The inhibition of eugenol on IAV replication occurred at 1–5 h p.i. **(** **Figure 3(G)** **)**.

Using MTT method, we also determined the influence of eugenol on the cell death after IAV infection. As shown in **(** **Figure 3(H)** **)**, after IAV infection, the viability of cell was significantly decreased, eugenol could significantly rescue the viability of cells infected with IAV. According to the previous reports that IAV infection induced autophagic cell death [7], [8], here we speculated that eugenol might inhibit autophagic cell death induced by IAV infection.

**Figure 5:**
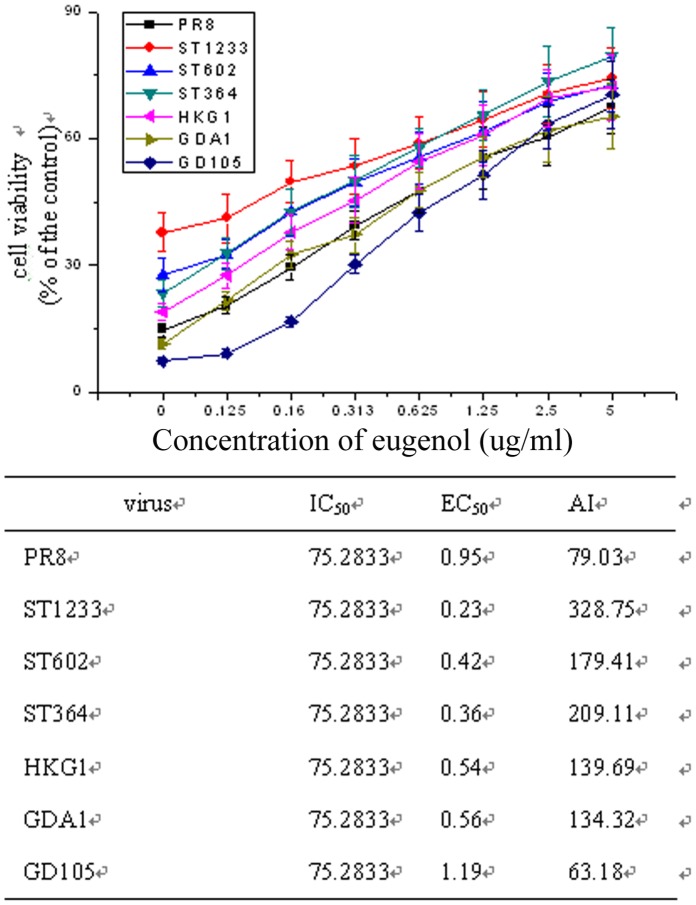
Antiviral activity of eugenol on A/PuertoRico/8/34 (H1N1, PR8), A/ShanTou/1233/06 (H1N1, ST1233), A/ShanTou/602/06 (H3N2, ST602), A/ShanTou/364/05 (H3N2, ST364), A/Quail/HongKong/G1/97 (H9N2, HKG1), A/Chicken/Guangdong/A1/03 (H9N2, GDA1) and A/Chicken/Guangdong/1/05 (H5N1, GD105) determined by the SRB method using CPE reduction. OD value was read at 562 nm. 0.5% DMSO was used in each group. Percent protection of test compounds (cell viability) was calculated according to the formula shown in Materials and methods. Concentration of 50% protection was defined as EC_50_. The antiviral index (AI) was defined as IC_50_/EC_50_. Data shown were the mean ± SD of three independent experiments.

Moreover, we also determined the effects of eugenol on other IAV subtypes including A/PuertoRico/8/34 (H1N1), A/ShanTou/1233/06 (H1N1), A/ShanTou/602/06 (H3N2), A/ShanTou/364/05 (H3N2), A/Quail/HongKong/G1/97 (H9N2), A/Chicken/Guangdong/A1/03 (H9N2) and A/Chicken/GD/1/05 (H5N1) by the SRB method using CPE reduction. The EC_50_ of eugenol to these IAV subtypes were 0.95, 0.23, 0.42, 0.36, 0.54, 0.56 and 1.19 µg/mL, respectively **(** **Figure 5** **)**.

### Eugenol could Inhibit the Oxidative Stress, the Activation of ERK1/2/p38MAPK and IKK/NF-κB Pathways Induced by IAV

**Figure 6:**
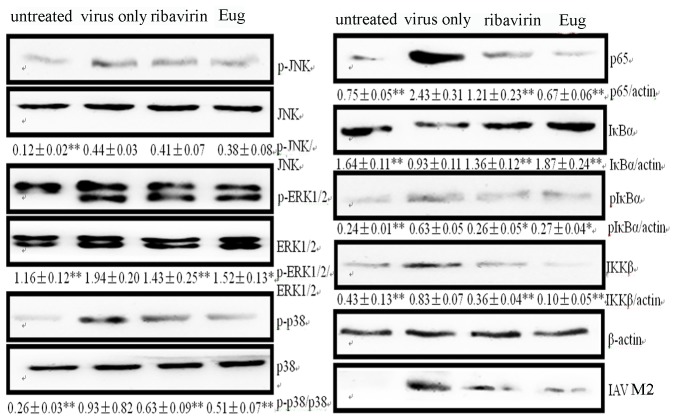
Effects of eugenol on the IAV-induced activation of ERK1/2, JNK1, p38MAPK and IKK/NF-κB signal pathways determined by Western blot. In the untreated group, A549 cells were not infected with IAV; in the negative control group (virus only), the cells were infected with IAV but not treated any drugs; in the ribavirin and Eug groups, the cells were infected, and treated with ribavirin (25 µg/mL) and eugenol (5 µg/mL), respectively. The infection time was 24 h, MOI = 0.001. The IAV strain was A/ShanTou/169/06(H1N1). The total gray value of each band was determined using Gel-Pro analyzer 6.0. The data were showed as the ratios of the target genes to β-actin. Data shown were the mean ± SD of three independent experiments. *P<0.05 and **P<0.01 vs the negative control group (virus only).

Up to now, there are two major doctrines, ‘cytokine storm’ and ‘oxidative stress’, which explain the mechanism of IAV-induced acute lung injury [26]. It is known that oxidative stress is essential for autophagy. ROS can activate autophagy via JNK1 [27], ERK1/2 [25], p38 and NF-κB [28] signal pathways. JNK1 can mediate the phosphorylation of Bcl-2 at residues T69, S70, and S87, ERK1/2 also phosphorylates Bcl-2, both of them disrupt the Beclin1- Bcl-2 complex [29]. Here we detected the influence of eugenol on oxidative stress and these signal pathways after IAV infection. As shown in **Table 1**, after IAV infection, MDA, NO and ROS were significantly increased, GSH, T-SOD, GR, and CAT were significantly decreased, and the cells displayed an obvious oxidative stress. Both of ribavirin (25 µg/mL) and eugenol (5 µg/mL) could significantly reverse these parameters. Correspondingly, IAV infection could activate the ERK1/2, JNK1, p38MAPK and IKK/NF-κB signal pathways, both of ribavirin (25 µg/mL) and eugenol (5 µg/mL) could significantly inhibit the activation of ERK1/2, p38MAPK and IKK/NF-κB signal pathways, but had no effect on JNK1 signal pathway (**Figure 6** **)**.

**Table 1:** Effects of eugenol on IAV-induced oxidative stress.

Goup	GSH(nmol GSH/g tissue)	MDA(nmol/mg prot)	NO(nmol/mg prot )	ROS(fold change to theuntreated group)
untreated	28.05±2.92**	0.057±0.013**	30.09±2.10**	1.00±0.00**
virus only	14.92±2.48	0.237±0.050	81.70±9.05	1.92±0.09
ribavirin	22.36±1.73*	0.141±0.047**	66.62±3.99*	1.64±0.08*
Eug	24.15±4.99**	0.086±0.016**	58.66±7.57**	1.32±0.07**
**Goup**	**T-SOD(U/mg prot**	**GR(nmol NADPH** **oxidized)**	**CAT(nmol H_2_O_2_ consumed/** **min/mg protein )**	**GSH-Px(10^3^U/mg prot)**
untreated	6.74±1.45**	96.99±7.34**	58.06±2.32**	2.13±0.27
virus only	2.73±0.64	51.51±5.96	28.75±7.30	2.20±0.20
ribavirin	4.15±0.94	80.48±3.58**	38.92±2.72*	2.71±0.21*
Eug	4.63±0.93*	89.65±3.69**	49.27±3.86**	3.15±0.33**

In the blank group (untreated), A549 cells were not infected with IAV (A/ShanTou/169/06(H1N1)). In the negative control group (virus only), A549 cells were infected, but not treated with any drugs. In the positive control (ribavirin) and Eug groups, A549 cells were infected, and treated with ribavirin (25 µg/ml) and eugenol (5 µg/mL), respectively. The infection time was 24 h, MOI = 0.001. Each value represents mean ± SD, n = 6.

* *P*<0.05 and

** *P*<0.01 *vs*. the negative control group (virus only).

### Eugenol could Inhibit Autophagy, ERK1/2/p38MAPK and IKK/NF-κB Pathways without IAV Infection and Antagonized the Effects of the Activators of these Signal Pathways

**Figure 7:**
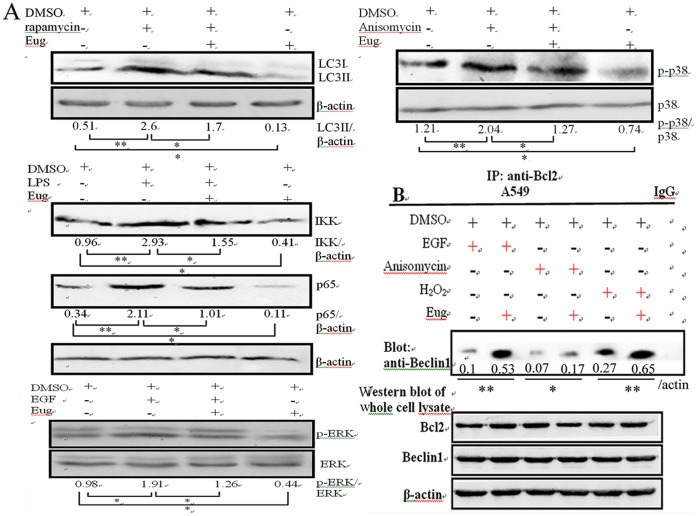
Antagonistic effect of eugenol against the activators of autophagy, ERK, p38 MAPK and IKK/NF-κB signal pathways without IAV infection. (A) Eugenol antagonized the effects of the activators of autophagy, ERK, p38 MAPK and IKK/NF-κB pathways determined by Western blot. A549 cells were seeded in 6-well plate for 24 h, then treated with or without eugenol (5 µg/mL) and the activators (autophagy activator 5 µM rapamycin, IKK/NF-κB activator 10 µM LPS, ERK activator 100 ng/ml EGF and p38/JNK activator 10 µM Anisomycin), after 24 h, the cells were collected and subjected to western blot using their corresponding antibodies. (B) Eugenol antagonized the promotions of the activators on the dissociation of Beclin1-Bcl2 heterodimer determined by co-IP assay. A549 cells were seeded in 6-well plate for 24 h, and then treated with or without eugenol (5 µg/mL) and the activators (100 ng/ml EGF, 10 µM Anisomycin and 100 µM H2O2), after 24 h, the cells were collected and subjected to co-IP assay. Data shown were the mean ± SD of three independent experiments. *P<0.05 and **P<0.01.

It has been reported that eugenol can inhibit the activation of ERK1/2, p38MAPK and IKK/NF-κB pathways in vivo [30] and in macrophages [31], here we determined the effects of eugenol on these pathways in A549 cells without IAV infection, and determined the antagonistic effect of eugenol on the effects of the activators of these signal pathways. As shown in **Figure 7(A)**, after treated with the activators of autophagy (5 µM rapamycin), IKK/NF-κB (10 µM LPS), ERK (100 ng/mL EGF) and p38/JNK (10 µM Anisomycin), the autophagy and the corresponding signal pathways were activated, eugenol could antagonize the effects of these activators. Moreover, without the treatments of these activators, eugenol also could inhibit autophagy and the activation of ERK1/2, p38MAPK and IKK/NF-κB pathways in A549 cells. In addition, eugenol also could antagonize the promotions of the activators (EGF, Anisomycin) and oxidant (H_2_O_2_) on the dissociation of Beclin1-Bcl2 heterodimer (**Figure 7(B)**). Accordingly, autophagy activator (rapamycin) could antagonize the antiviral ability of eugenol in a concentration dependent manner determined by the SRB method using CPE reduction and by plaque inhibition assay. (**Figure S7**). We speculated that the ability of eugenol inhibiting IAV replication was related to its inhibition of autophagy.

### Eugenol could Inhibit the Expressions of Autophagic Genes Induced by IAV

**Figure 8:**
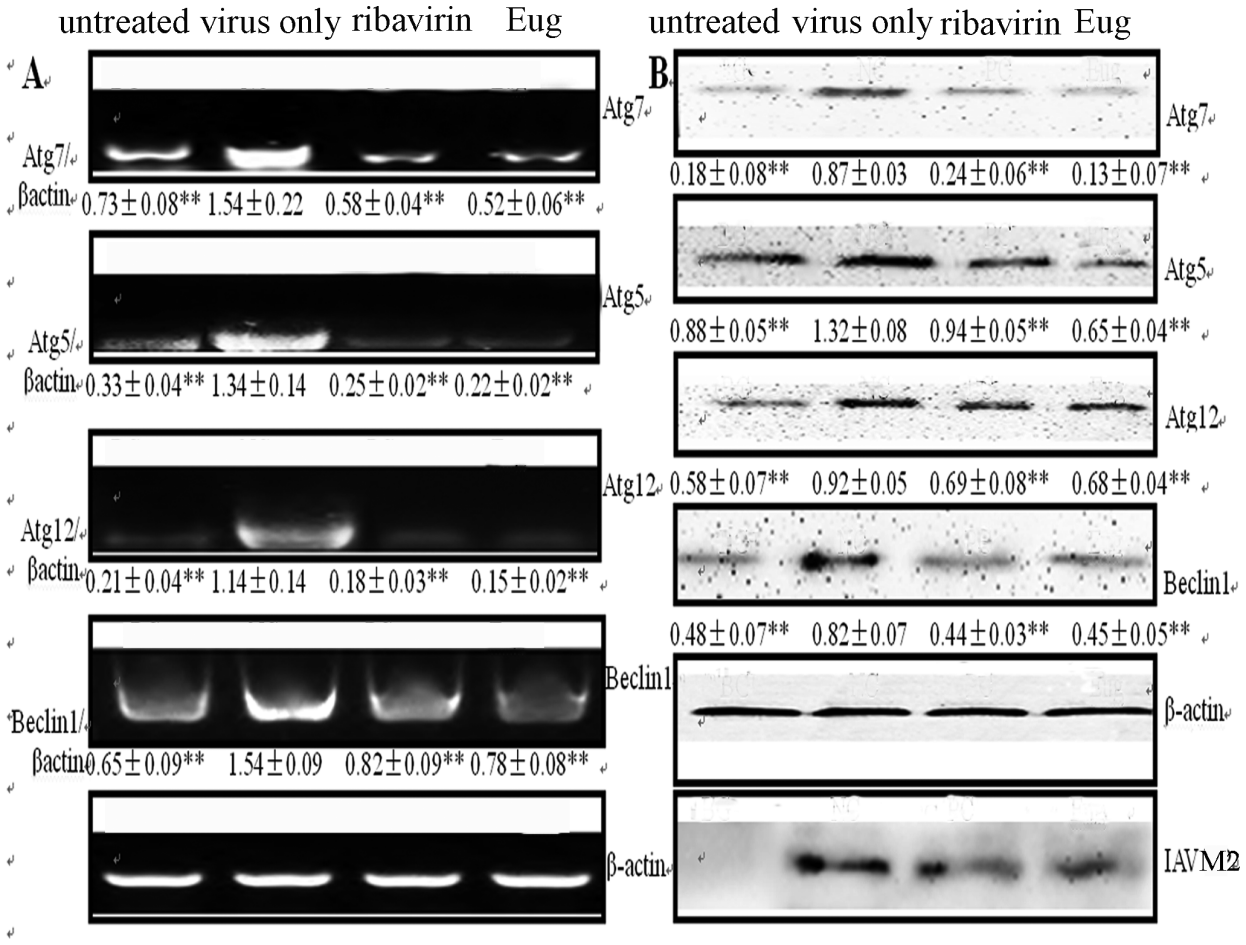
Eugenol inhibited the expression of autophagic genes after IAV infection (A/ShanTou/169/06(H1N1)). (A) RT-PCR assay. (B) Western blot assay. The treatments of untreated, virus only, ribavirin and Eug groups were same with those in Figure 6. The infection time was 24 h, MOI = 0.001. Data shown were the mean ± SD of three independent experiments. *P<0.05 and **P<0.01 vs the negative control group (virus only).

It is reported that the activation of JNK1, ERK1/2 and IKK/NF-κB pathways can increase the expressions of autophagic genes [16], [17], [32], which may lead to the increase of autophagic flux. Here we investigated the influence of eugenol on the expression of Atg5, Atg7, Atg12, and Beclin1. As shown in **Figure 8**, the expressions of Atg5, Atg7, Atg12 and Beclin1 were significantly increased after IAV infection, and significantly inhibited by ribavirin and eugenol at both mRNA and protein levels.

### Eugenol could Inhibit the Releases of Cytokines Induced by IAV

**Figure 9:**
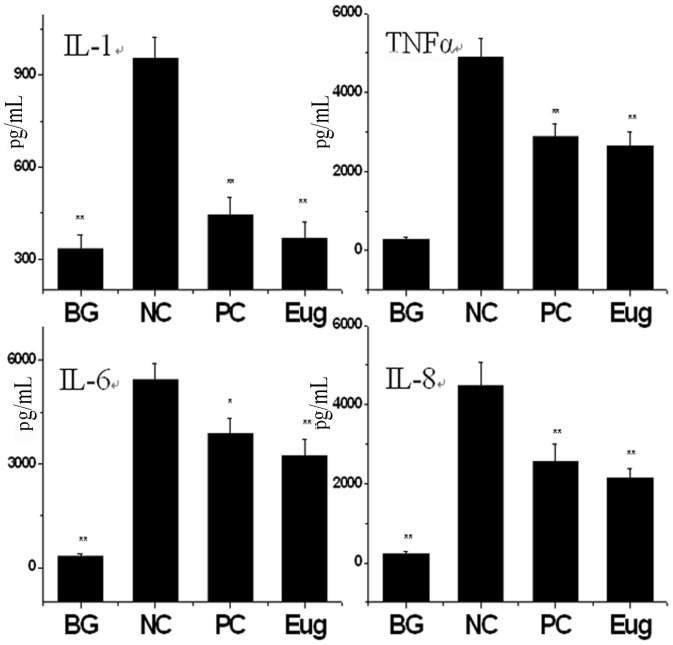
Eugenol inhibited the release of cytokines induced by IAV (A/ShanTou/169/06 (H1N1)). The treatments of untreated, virus only, ribavirin and Eug groups were same with those in Figure 6. The infection time was 24 h, MOI = 0.001. Data shown were the mean ± SD of three independent experiments. *P<0.05, **P<0.01 vs the negative control group (virus only).

It is reported that autophagy can regulate the release of cytokines [33], and cytokine storm is an important pathogenesis of IAV-induced acute lung injury. In above experiment, we found that eugenol could inhibit the IAV-induced activation of IKK/NF-κB pathway which was an important pathway to control the release of proinflammatory cytokine. Here we also determined the influence of eugenol on the releases of IL-1, TNF-α, IL-6 and IL-8. As shown in **Figure 9**, after IAV infection, the levels of these cytokines were significantly increased, whereas both of ribavirin and eugenol significantly inhibited the releases of these cytokines.

## Discussion

Up to now, there are two anti-IAV drug screening strategies: virus-based and cell-based drug screening models. The former mainly targets the neuraminidase, M2 ion channel and RNA-dependent RNA polymerase [34], [35], and the latter mainly targets the IAV-induced cytopathic effect (CPE) [36], [37]. Influenza A virus possesses high genetic variability, virus-based drug screening models often lead to the inevitable selection of drug-resistant viral mutants and drug-resistant variants can rapidly generate. Hunan genetic variability is very low, so cell-based drug screening models often decrease these resistant mutants [38]. Now, targeting human protein which is essential for viral replication has become an important strategy for developing new antiviral drugs [39]. In our research, we first established a screening model to screen novel autophagy inhibitors based on the inhibition of the dissociation of Beclin1-Bcl2 heterodimer, and according to the previous reports that autophagy is essential for IAV replication or induces autophagic cell death which results in acute lung injury [6], [7], [8], we then detect the anti-IAV activity of these autophagy inhibitors. Because autophagy is a highly conserved process in all eukaryotic cells, so our drug screening model may lead to the discovery of novel antiviral drugs that resist the development of drug-resistant virus strains.

Though there are many reports that IAV infection can enhance autophagic flux, some researchers think that the accumulation of autophagosome after IAV infection is not the consequence of a general enhancement of autophagy, but is only the block of autophagosome maturation by the binding of M2 to Beclin1 [40]. We do not support this point. As aforementioned, IAV involves in autophagy by its M2 protein binding with Beclin1, but there are at least three kinds of Beclin1 complexes. The Atg14L complex localizes to the autophagosome and ER, and functions in autophagosome formation. The UVRAG complex functions in autophagosome maturation. The Rubicon complex localizes at the endosome/lysosome and suppresses autophagosome maturation [10]. We think that IAV M2 can interact with all of these three complexes, it not only influences the autophagosome maturation, but also influences the autophagosome initiation and formation. In fact, there are many proteins and signal pathways can upregulate the expression of autophagic genes and increase autophagic flux. HMGB1 can increase the expression of Beclin1, Vps34 and UVRAG and enhance autophagic flux [15]. ROS can increase the expression of Atg5 and Beclin1 and enhance autophagic flux [27], [41]. NF-κB p65 directly binds the Beclin1 promoter and upregulates the expression of Beclin1 [42]. IKK can promote the autophagic pathway via the canonical AMPK/mTOR pathway and JNK1 pathway [16], [17]. IAV infection can activate these proteins and these signal pathways, so we think the increase of autophagic flux induced by IAV infection plays an important role in the IAV-induced accumulation of autophagosome, and effectively controlling the autophagic flux is important for inhibiting autophagic cell death and acute lung injury induced by IAV. This is the reason that we select the interaction of Beclin1 and Bcl2, which can at least in part control the autophagic flux, as our drug screening target.

**Figure 10:**
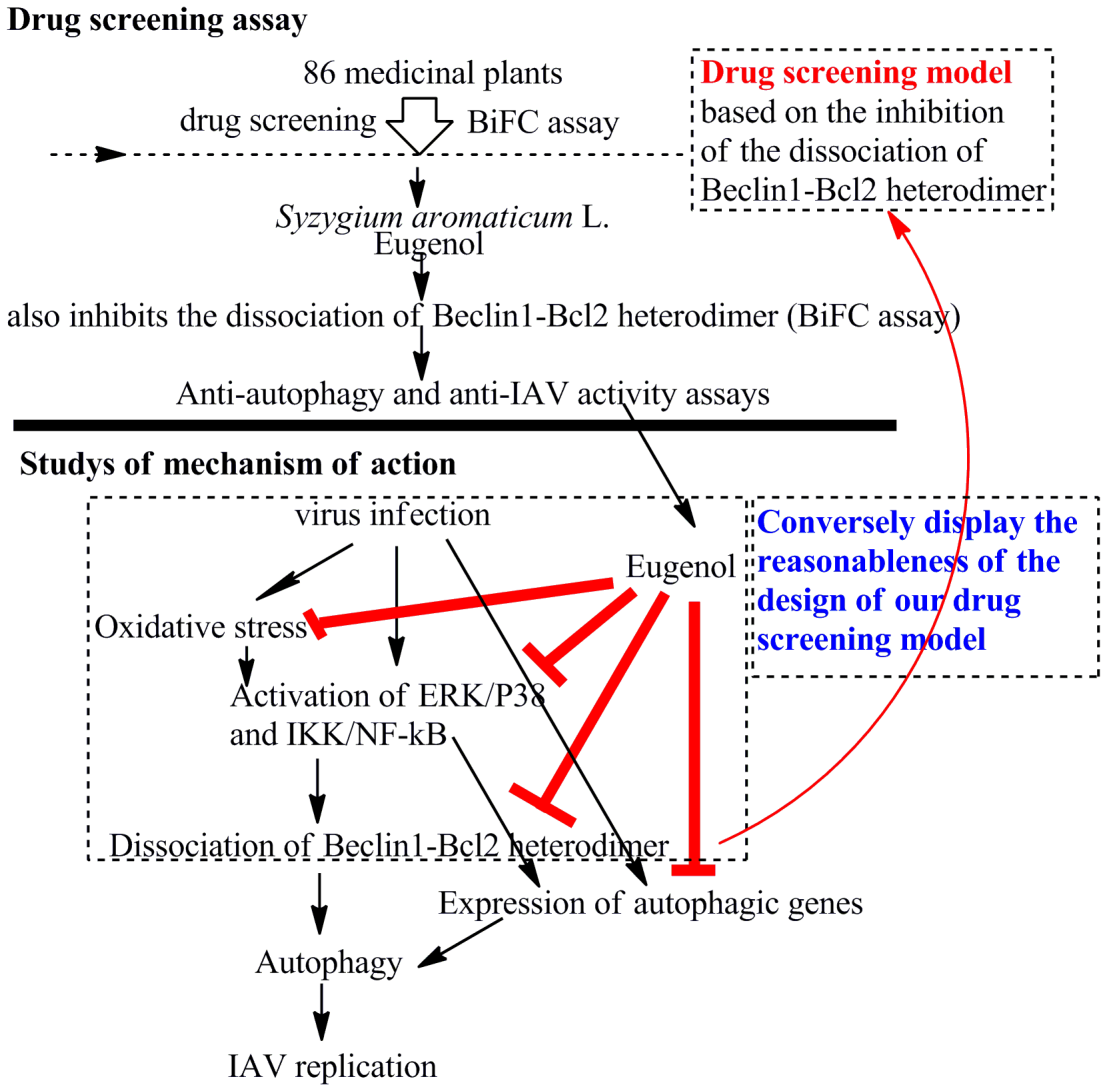
The design of our experiment and the mechanism of action of eugenol. Eighty six traditional Chinese medicines were screened by our drug screening model which was based on the inhibition of the dissociation of Beclin1-Bcl2 heterodimer, and *Syzygium aromaticum* L. was found to have the best activity. We purchased eugenol, the major active compound of *Syzygium aromaticum* L. and found it also inhibited the dissociation of Beclin1-Bcl2 heterodimer. We then detected the anti-autophagy and anti-IAV activity of eugenol. Next we explored the mechanism of action of eugenol. Eugenol could inhibit the oxidative stress and the activation of ERK/JNK/p38 MAPK and IKK/NF-κB pathways induced by IAV infection, both of them were important regulators of the dissociation of Beclin1-Bcl2 heterodimer, and thus conversely displayed the reasonableness of the design of our drug screening model. Moreover, eugenol also inhibited the expression of autophagic genes. Eventually, eugenol inhibited autophagy and impaired IAV replication.

Using this model, we have screened 86 examples of traditional herb, find 15 examples have significant (*P*<0.05) activity, and *Syzygium aromaticum* L. shows the best activity. We then pick out eugenol, detect its anti-IAV activity, explore its mechanism of action, and simultaneously display the reasonableness of the design of our screening model. We find eugenol can inhibit the dissociation of Beclin1-Bcl2 heterodimer and autophagy, ameliorates the oxidative stress, inhibits the activation of ERK, p38 MAPK and IKK/NF-κB signal pathways, and inhibits the expression of autophagic genes. We also find that without IAV infection, eugenol also can inhibit autophagy and the activation of ERK1/2, p38MAPK and IKK/NF-κB pathways, and antagonizes the effects of the activators of these pathways. We speculate that the mechanism of action of eugenol may be that eugenol inhibits the oxidative stress, the activation of ERK1/2, p38MAPK and IKK/NF-κB pathways, then inhibits the dissociation of Beclin1-Bcl2 heterodimer, additionally, eugenol also inhibits the expressions of autophagic genes, such as Beclin1, and finally inhibits autophagy and impairs IAV replication. This conversely displays the reasonableness of the design of our screening model (**Figure 10**).

NF-κB signal is an important regulator of the production of cytokines. Autophagy inducers can promote the phosphorylation of IKKα/β on serines 177/181 and of IKKγ/NEMO on serine 376, and trigger the activation of the IKK complex and the IκB degradation [16], [17]. In our experiment, eugenol shows the ability to inhibit the activation of IKK/NF-κB, so we also determine the levels of cytokines and find eugenol also can inhibit the release of cytokines, which may ameliorate the ‘cytokine storm’ induced by IAV.

In conclusion, we have established a drug screening model based on the inhibition of the dissociation of Beclin1-Bcl2 heterodimer, an important regulator of autophagy. Using this model, we screen 86 examples of traditional Chinese medicine and find 15 examples can significantly inhibit the dissociation of Beclin1-Bcl2 heterodimer. We then detect the anti-autophagy and anti-IAV activity, and explore the mechanism of action of eugenol, and show that eugenol is a promising inhibitor for autophagy and IAV infection.

## Materials and Methods

### Medicinal Plants and Compound

Medicinal plants were collected from the Yulin medicinal market (Guangxi, China). The specimens were deposited in our lab. Eugenol (4-allyl-2-methoxyphenol, Product ID: E51791, purity >98%) was purchased from Sigma-Aldrich (Shanghai) Trading Co., Ltd.

### Viruses and Cells Cytotoxicity Assay

IAV subtypes A/ShanTou/169/06(H1N1), A/PuertoRico/8/34 (H1N1), A/ShanTou/1233/06 (H1N1), A/ShanTou/602/06 (H3N2), A/ShanTou/364/05 (H3N2), A/Quail/HongKong/G1/97 (H9N2), A/Chicken/Guangdong/A1/03 (H9N2) and A/Chicken/GD/1/05 (H5N1) were used. The virus stocks were prepared in MDCK cells or 10-day-old embryonating eggs. The virus titers were determined by a plaque assay [19]. The cytotoxicity of all extracts and eugenol was determined by MTT method on A549 and MDCK cells [43]. The concentration required to decrease cell viability by 50% (IC_50_) was calculated. The maximal concentration without cytotoxicity was used as the optimal concentration.

### Plasmids Construction

To construct the BiFC plasmids, two segments from a red fluorescent protein (GenBank: HQ423140.1), corresponding to amino acids 1 to 159 and 160 to 262, respectively, were inserted into a pcDNA3.0 plasmid and named pMN and pMC, respectively. Human Bcl2 (NM_000633.2) were inserted into pMN and named pMN-Bcl2. Human Beclin1 (NM_003766.3) was inserted into pMC and named pMC-Beclin. To construct eukaryotic expression plasmid, human HMGB1 (NM_002128.4) and MyD88 (NM_001172567.1) were inserted into pcDNA3.0 and named pcDNA-HMGB1 and pcDNA-MyD88, respectively. To construct a pEGFP-LC3 plasmid, human LC3B (NM_022818.4) was inserted into a pEGFP-C1 plasmid. All constructs were verified by double enzyme digestion and DNA sequencing.

### Drug Screening Assay (BiFC Assay)

A549 cells were seeded in 96-well plate for 24 h, after cotransfection with the corresponding plasmids for 6 h, IAV (MOI = 2.0) and test drugs at the maximal no-cytotoxicity concentration were added, and incubated for 8 h, after treatment at 4°C for 1h, the fluorescence intensities (FI) were measured at 610 nm after excitation at 587 nm using a microplate reader (Tecan infinite M1000) and calculated as following:
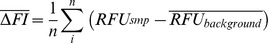


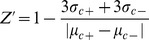



The Z’-factor was a statistical parameter to quantify the suitability of a particular assay for use in a high-throughput screen [19]. The notations σ_c+_ and σ_c-_ were the standard deviations of the negative control (NC) and blank group (BG), respectively, and µ_c+_ and µ_c−_ were the average values of the NC and BG groups, respectively. To observe the results of BiFC assay, A549 cells were seeded onto glass cover slip in a 12-well plate, and visualized under an upright fluorescence microscope (Nikon Eclipse 90i).

### Plaque Inhibition and Time-of-addition Assay

Plaque inhibition assay was performed on MDCK cells [19]. Multiplicity of infection (MOI) was 0.001. The incubation time after absorption was 48 h. The supernatants were collected and the virus titers were determined by a plaque assay [19]. The concentration required to inhibit virus titer by 50% (EC_50_) was calculated. Antiviral index (AI) = IC_50_/EC_50_. Time-of-addition assay consisted of four tests [44]: 1) Before infection, the virus was pretreated by eugenol (5 µg/mL); 2) Before infection, the cells were pretreated by eugenol (5 µg/mL); 3) Eugenol was added during viral adsorption; 4) Eugenol (5 µg/mL) was added at different time points after virus challenge. MOI = 2.0. The incubation time after absorption was 12 h. The other performances were same with the plaque inhibition assay.

### TCID50 and Antiviral Assay by the SRB Method Using CPE Reduction

IAV stock solution was diluted with DMEM containing 2.5 µg/mL trypsin and 3.2% BSA in serial dilutions, after incubation with MDCK cells for 48 h, the TCID_50_ was calculated following the method of Reed and Muench. Antiviral activities of test compounds were evaluated by the sulforhodamine B (SRB) method using CPE reduction [45]. Briefly, MDCK cells were seeded in 96-well plate. 0.09 mL of virus suspension (50 TCID_50_) and 0.01 mL medium containing various concentrations of test compounds were added. At 48 h, after washing, 100 µl −20°C 70% acetone was added. After removing acetone, the plates were dried, and added 100 µl 0.4% (w/v) SRB, after washing, the plates were dried and added 100 µl 10 mM Tris-base solution. OD was read at 562 nm. Three wells were used each for the negative (virus-infected non-drug-treated) and the mock controls (non-infected non-drug-treated). 0.5%DMSO was used in each group. Percent protection of test compounds (cell viability) was calculated as following, the concentration of 50% protection was defined as the EC_50_.
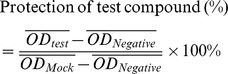



### EGFP-LC3II Assay

After transfection with the pEGFP-LC3 plasmid for 6 h, A549 cells were infected with IAV (MOI = 2.0) and treated with eugenol (5 µg/mL), at 8 h p.i., the cells were visualized using an invert fluorescence microscope (10×40), the percentages of cells containing EGFP-LC3 dots to cells expressing EGFP were calculated in 10 fields chosen at random from three independent experiments.

### Reverse-transcription (RT-PCR)

Extraction of RNA and RT-PCR reactions were performed according to the protocols of the TRIzol® reagent kit and the RT-PCR kit (Invitrogen). PCR products were electrophoresed in a 1% agarose gel and visualized on an UV-transilluminator.

### Western Blotting and Co-Immunoprecipitation (co-IP) Assay

Anti-LC3B, anti-Beclin1, anti-Atg5, anti-Atg7, anti-Atg12, anti-Bcl2, anti-IAV M2, anti-p65, anti-IκBα, anti-pIκBα, anti-IKKβ, anti-pJNK, anti-JNK, anti-pERK, anti-ERK, anti-p-p38, anti-p38 and anti-β-actin antibodies were purchased from Cell Signaling Technology® Inc. Company. To detect NF-κB p65, the nucleic protein was extracted. The Western blotting assay was performed as previously reported [19]. The influence of drugs on the dissociation of Beclin1-Bcl2 heterodimer and of Beclin1-IAV M2 heterodimer were detect by co-IP assay, A549 cells were seeded into a 6-well plate for 24 h, after cotransfection with corresponding plasmids for 6 h, the drugs were added, and the cells were collected after 24 h. The interactions were determined following the instrument of the Co-Immunoprecipitation Kit (Thermo scientific, #23600). Normal rabbit IgG was used as a control.

### Antioxidant Assay

Levels of reduced glutathione (GSH), malondialdehyde (MDA), NO, total superoxide dismutase (T-SOD), glutathione reductase (GR), catalase (CAT) and glutathione peroxidase (GSH-Px) were determined using commercially available kits (Jiancheng Bioengineering Institute, Nanjing, China). The level of intracellular ROS was determined using 2′,7′- dichlorofluorescein diacetate (DCFH-DA) method. The reactive oxygen species (ROS) assay kit (Cat#: S0033) was purchased form Beyotime Institute of Biotechnology, China, and performed according to the directions.

### ELISA Assay

Cell culture supernatants were collected and frozen at −80°C. The levels of IL-1, IL-6, IL-8 and TNF-α were determined using commercially available kits (Boster Biotech. Inc., Wuhan, China).

### Statistical Analysis

Data were expressed as means ± SD. Statistical significance was determined using the SPSS13.0 software.

## Supporting Information

Figure S1 **The regulation of autophagy and the involvement of IAV.** The precursors of autophagosome (phagophore) is mainly from endoplasmic reticulum and golgiosome **(i)**; Phagophore captures cytoplasmic targets (cytosol, organelles such as mitochondria, pathogens) **(ii)**; Phagophore enlarges assisted by Atg5–Atg12/Atg16 protein complex and LC3-II, warps around its cytoplasmic target and closes to form a double membrane autophagosome **(iii)**; Autophagosome fuses with lysosomes **(iv)** and converts into an acidified, hydrolytic organelle termed autolysosome **(v)**; finally the captured cargo is degraded **(vi)**. In autophagy induction, the kinase mTOR is a critical negative regulator. Upstream of mTOR, the TSC1/2 complex accepts the regulations of PI3KCI/Akt, LKB1/AMPK, MEM/ERK and HIF-1/REDD1 signal pathways, and negatively regulates the mTOR activity through Rheb. mTOR phosphorylates and negatively regulates Atg13-ULK1- FIP200 complex which is an initial complex of autophagy. Downstream of mTOR, Beclin1 is a very important protein required for autophagy which exists in three kinds of complexes. Beclin1 complex I (Atg14L complex) consists of Atg14L, Beclin 1, Vps34 and p150, localizes to the autophagosome and ER, and is required for the induction of autophagy; Beclin1 complex II (UVRAG complex) consists of UVRAG, Beclin1, Vps34 and p150, and functions in autophagosome maturation; Beclin1 complex III (Rubicon complex) consists of Rubicon, UVRAG, Beclin1, Vps34 and p150, and inhibits the autophagosome maturation. In addition, many proteins, such as Bif-1, Ambra1, nPIST, VMP1, SLAM, PINK1 and Survivin, interact with Beclin1 and regulate the function of Beclin1. Among Beclin1-binding proteins, Bcl2 functions as an important autophagy inhibitor. Beclin1 is tightly bound and regulated by Bcl2, the dissociation of Beclin 1 from Bcl2 is crucial for autophagy and regulated by some signal pathways, such as DAPK, IKK, PKC, ERK, JNK1, TLRs/MyD88/TRIF/TRAF6, and some proteins, such as HMGB1, MyD88, TRIF, BNIP3, Bad, Noxa, Puma, BimEL, Bik, Bif-1, Ambra1, nPIST, VMP1, SLAM, PINK1 and Survivin. We speculates that IAV may involve in autophagy by **(1)** TLRs/Nox2 NADPH oxidase promoting the initiation of autophagy, **(2)** oxidative stress, Atg4 and Atg7 influencing the formation of atg12-atg5/atg16 complex and LC3II, **(3)** the activations of the IKK, PKC, JNK1, ERK and TLRs/MyD88/TRIF/TRAF6 signal pathways promoting the dissociation of Beclin1 from Bcl2, **(4)** IAV M2 protein binding with Beclin1, and **(5)** promoting the expression of autophagic genes.
(DOC)

Figure S2 **Reconstitution of RFP and the influence of HMGB1 and MyD88 on the Beclin1-Bcl2 heterodimer. (A)** Reconstitution of RFP. A549 cells were cotransfected with pMN-Bcl2 and pMC-Beclin1, after 8 h, the cotransfected cells appeared a lot of red fluorescence. These graphs were corresponding to **Figure 1B** **c** in text. **(B)** The influence of HMGB1 and MyD88 on the Beclin1-Bcl2 heterodimer. Beclin1-binding proteins HMGB1 and MyD88 were expected to disrupt the Beclin1-Bcl2 heterodimer, after cotransfection for 8 h, the cells were visualized, These graphs were corresponding to **Figure 1C** **a**, **b and c** in text. The ratios of RFP-positive cells were calculated in 5 fields chosen at random from three independent experiments, the data were shown as mean±SD, Data shown were the mean ± SD. *P<0.05 and **P<0.01 vs. the untreated group.
(DOC)

Figure S3 **The influence of ERK1/2 inhibitor (U0126, 10 µM), ERK1/2 activator (EGF, 100** **ng/ml), JNK/p38 inhibitor (SB203580, 40 µM), p38 MAPK activator (anisomycin, 10 µM), antioxidant (NAC, 2 mM) and oxidant (H2O2, 100 µM) on the dissociation of Beclin1-Bcl2 heterodimer.** After cotransfection, A549 cells were treated with these inhibitors and activators, after 8 h, the cells were visualized, These graphs were corresponding to Figure 1D a, b, c, d, e and f in text. The ratios of RFP-positive cells were calculated in 5 fields chosen at random from three independent experiments. Data shown were the mean ± SD. *P<0.05, **P<0.01.
(DOC)

Figure S4 **The influences of eugenol on the Beclin1-Bcl2 heterodimer with IAV infection.** After cotransfection with pMC-Beclin1 and pMN-Bcl2, A549 cells were infected with IAV (A/ShanTou/169/06(H1N1)) (MOI = 2.0) and treated with ribavirin (25 µg/ml) and eugenol (5 µg/mL, after 8 h, the cells were visualized, these graphs were corresponding to **Figure 2A** **a, b, c and d** in text. The ratios of RFP-positive cells were calculated in 5 fields chosen at random from three independent experiments. Data shown were the mean ± SD. *P<0.05, **P<0.01 vs. the virus only group.
(DOC)

Figure S5 **The influences of eugenol on the Beclin1-Bcl2 heterodimer without IAV infection.** After cotransfection with pMC-Beclin1 and pMN-Bcl2, A549 cells were not infected with IAV but directly treated with ribavirin (25 µg/ml) and eugenol (5 µg/mL), after 8 h, the cells were visualized, these graphs were corresponding to **Figure 2B** **a, b and c** in text. The ratios of RFP-positive cells were calculated in 5 fields chosen at random from three independent experiments. Data shown were the mean ± SD. *P<0.05, **P<0.01 vs the untreated group.
(DOC)

Figure S6 **Eugenol inhibited the accumulation of autophagosomes determined by EGFP-LC3 assay.** A549 cells were transfected with the pEGFP-LC3 plasmid. In the untreated group, A549 cells were not infected with IAV. In the virus only treated group, A549 cells were infected but not treated with any drugs. In the ribavirin and Eug treated groups, A549 cells were infected and treated with ribavirin (25 µg/ml) and eugenol (5 µg/mL), respectively. The incubation time was 8 h, MOI = 2.0, The percentage of cells containing EGFP-LC3 dots to cells expressing EGFP was calculated in 10 fields chosen at random. The graphs were obtained from an inverted fluorescence microscope (10×40 and 10×100). Data shown were the mean ± SD of three independent experiments. *P<0.05 and **P<0.01 vs. NC.
(DOC)

Figure S7 **Rapamycin antagonized the anti-IAV activity of eugenol.** A549 cells were infected (MOI = 0.001) and treated with eugenol (5 µg/mL), and simultaneously treated with or without various concentrations of rapamycin (0, 0.625, 1.25, 2.5, 5, 10 µM), after 48 h, the antiviral activity (cell viability) was determined by the SRB method using CPE reduction (**A**). In another experiment, the supernatants were collected and the virus titers were determined by a plaque assay on MDCK cells (**B**). Additionally, rapamycin could promote the replication of IAV (**C**). Data shown were the mean ± SD of three independent experiments. *p<0.05, **p<0.01.*vs* the virus+eugenol.
(DOC)

Table S1 **Influence of plant extracts on the dissociation of the Beclin1-Bcl2 heterodimer (fold change).** A549 cells were seeded in 96-well plate for 24 h, then cotransfected with pMC-Beclin1 and pMN-Bcl2 plasmids, after 6 h, in the blank group (BG, untreated), A549 cells were not infected with IAV; in the negative control (NC, virus only) group, A549 cells were infected, but not treated with any drugs; in the positive control (PC, ribavirin) and test drug groups, A549 cells were infected, and treated with ribavirin (25 µg/ml) and test drugs at the maximum no-cytotoxicity concentrations, respectively. MOI = 2.0, the incubation times were 8 h. The FI was determined at 610 nm after excitation at 587 nm using a microplate reader (Tecan infinite M1000). All numbers were expressed as fold change relative to the negative control (NC, virus only). Data shown were mean ± SD from three independent experiments performed in triplicate.*P<0.05, **P<0.01 *vs.* the negative control (NC, virus only).
(DOC)
