# Supplementary material for: Drug Screening for Autophagy Inhibitors Based on the Dissociation of Beclin1-Bcl2 Complex Using BiFC Technique and Mechanism of Eugenol on Anti-Influenza A Virus Activity
Source: PLoS One. 2013 Apr 16;8(4):e61026. doi: 10.1371/journal.pone.0061026 (PMC3628889; doi:10.1371/journal.pone.0061026)
Supplement: Figure S1 — The regulation of autophagy and the involvement of IAV. The precursors of autophagosome (phagophore) is mainly from endoplasmic reticulum and golgiosome (i); Phagophore captures cytoplasmic targets (cytosol, organelles such as mitochondria, pathogens) (ii); Phagophore enlarges assisted by Atg5–Atg12/Atg16 protein complex and LC3-II, warps around its cytoplasmic target and closes to form a double membrane autophagosome (iii); Autophagosome fuses with lysosomes (iv) and converts into an acidified, hydrolytic organelle termed autolysosome (v); finally the captured cargo is degraded (vi). In autophagy induction, the kinase mTOR is a critical negative regulator. Upstream of mTOR, the TSC1/2 complex accepts the regulations of PI3KCI/Akt, LKB1/AMPK, MEM/ERK and HIF-1/REDD1 signal pathways, and negatively regulates the mTOR activity through Rheb. mTOR phosphorylates and negatively regulates Atg13-ULK1- FIP200 complex which is an initial complex of autophagy. Downstream of mTOR, Beclin1 is a very important protein required for autophagy which exists in three kinds of complexes. Beclin1 complex I (Atg14L complex) consists of Atg14L, Beclin 1, Vps34 and p150, localizes to the autophagosome and ER, and is required for the induction of autophagy; Beclin1 complex II (UVRAG complex) consists of UVRAG, Beclin1, Vps34 and p150, and functions in autophagosome maturation; Beclin1 complex III (Rubicon complex) consists of Rubicon, UVRAG, Beclin1, Vps34 and p150, and inhibits the autophagosome maturation. In addition, many proteins, such as Bif-1, Ambra1, nPIST, VMP1, SLAM, PINK1 and Survivin, interact with Beclin1 and regulate the function of Beclin1. Among Beclin1-binding proteins, Bcl2 functions as an important autophagy inhibitor. Beclin1 is tightly bound and regulated by Bcl2, the dissociation of Beclin 1 from Bcl2 is crucial for autophagy and regulated by some signal pathways, such as DAPK, IKK, PKC, ERK, JNK1, TLRs/MyD88/TRIF/TRAF6, and some proteins, such as HMGB1, MyD88, TRIF, [file pone.0061026.s001.doc]

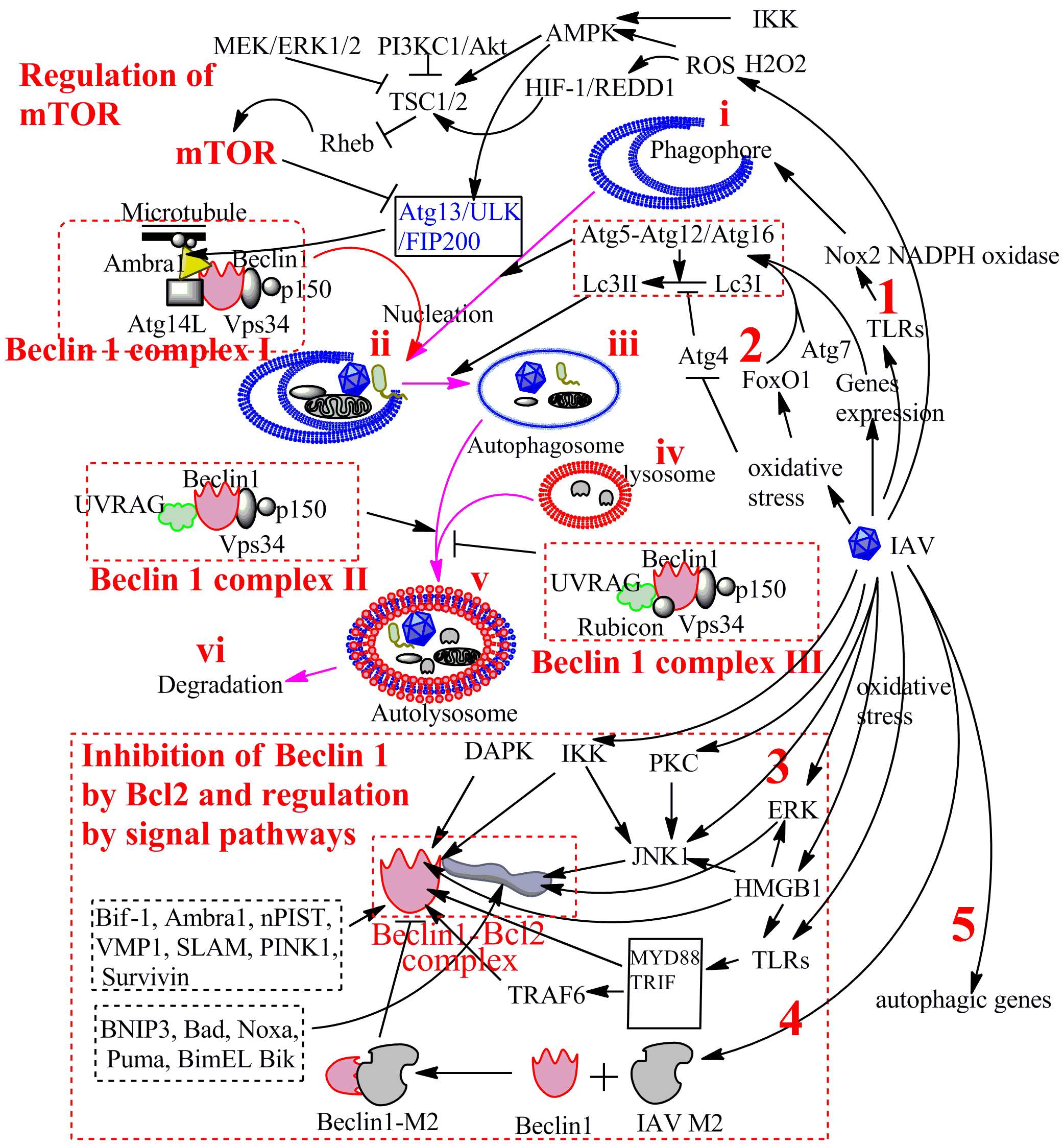


**Figure S1. The regulation of autophagy and the involvement of IAV**. The precursors of autophagosome (phagophore) is mainly from endoplasmic reticulum and golgiosome **(i)**; Phagophore captures cytoplasmic targets (cytosol, organelles such as mitochondria, pathogens) **(ii)**; Phagophore enlarges assisted by Atg5–Atg12/Atg16 protein complex and LC3-II, warps around its cytoplasmic target and closes to form a double membrane autophagosome **(iii)**; Autophagosome fuses with lysosomes **(iv)** and converts into an acidified, hydrolytic organelle termed autolysosome **(v)**; finally the captured cargo is degraded **(vi)**.

In autophagy induction, the kinase mTOR is a critical negative regulator. Upstream of mTOR, the TSC1/2 complex accepts the regulations of PI3KCI/Akt, LKB1/AMPK, MEM/ERK and HIF-1/REDD1 signal pathways, and negatively regulates the mTOR activity through Rheb. mTOR phosphorylates and negatively regulates Atg13-ULK1- FIP200 complex which is an initial complex of autophagy.

Downstream of mTOR, Beclin1 is a very important protein required for autophagy which exists in three kinds of complexes. Beclin1 complex I (Atg14L complex) consists of Atg14L, Beclin 1, Vps34 and p150, localizes to the autophagosome and ER, and is required for the induction of autophagy; Beclin1 complex II (UVRAG complex) consists of UVRAG, Beclin1, Vps34 and p150, and functions in autophagosome maturation; Beclin1 complex III (Rubicon complex) consists of Rubicon, UVRAG, Beclin1, Vps34 and p150, and inhibits the autophagosome maturation. In addition, many proteins, such as Bif-1, Ambra1, nPIST, VMP1, SLAM, PINK1 and Survivin, interact with Beclin1 and regulate the function of Beclin1.

Among Beclin1-binding proteins, Bcl2 functions as an important autophagy inhibitor. Beclin1 is tightly bound and regulated by Bcl2, the dissociation of Beclin 1 from Bcl2 is crucial for autophagy and regulated by some signal pathways, such as DAPK, IKK, PKC, ERK, JNK1, TLRs/MyD88/TRIF/TRAF6, and some proteins, such as HMGB1, MyD88, TRIF, BNIP3, Bad, Noxa, Puma, BimEL, Bik, Bif-1, Ambra1, nPIST, VMP1, SLAM, PINK1 and Survivin.

We speculates that IAV may involve in autophagy by **(1)** TLRs/Nox2 NADPH oxidase promoting the initiation of autophagy, **(2)** oxidative stress, Atg4 and Atg7 influencing the formation of atg12-atg5/atg16 complex and LC3II, **(3)** the activations of the IKK, PKC, JNK1, ERK and TLRs/MyD88 /TRIF/TRAF6 signal pathways promoting the dissociation of Beclin1 from Bcl2, **(4)** IAV M2 protein binding with Beclin1, and **(5)** promoting the expression of autophagic genes.
