# Supplementary material for: Drug Screening for Autophagy Inhibitors Based on the Dissociation of Beclin1-Bcl2 Complex Using BiFC Technique and Mechanism of Eugenol on Anti-Influenza A Virus Activity
Source: PLoS One. 2013 Apr 16;8(4):e61026. doi: 10.1371/journal.pone.0061026 (PMC3628889; doi:10.1371/journal.pone.0061026)
Supplement: Figure S2 — Reconstitution of RFP and the influence of HMGB1 and MyD88 on the Beclin1-Bcl2 heterodimer. (A) Reconstitution of RFP. A549 cells were cotransfected with pMN-Bcl2 and pMC-Beclin1, after 8 h, the cotransfected cells appeared a lot of red fluorescence. These graphs were corresponding to Figure 1B c in text. (B) The influence of HMGB1 and MyD88 on the Beclin1-Bcl2 heterodimer. Beclin1-binding proteins HMGB1 and MyD88 were expected to disrupt the Beclin1-Bcl2 heterodimer, after cotransfection for 8 h, the cells were visualized, These graphs were corresponding to Figure 1C a, b and c in text. The ratios of RFP-positive cells were calculated in 5 fields chosen at random from three independent experiments, the data were shown as mean±SD, Data shown were the mean ± SD. *P<0.05 and **P<0.01 vs. the untreated group. (DOC) [file pone.0061026.s002.doc]

**B**

**A**


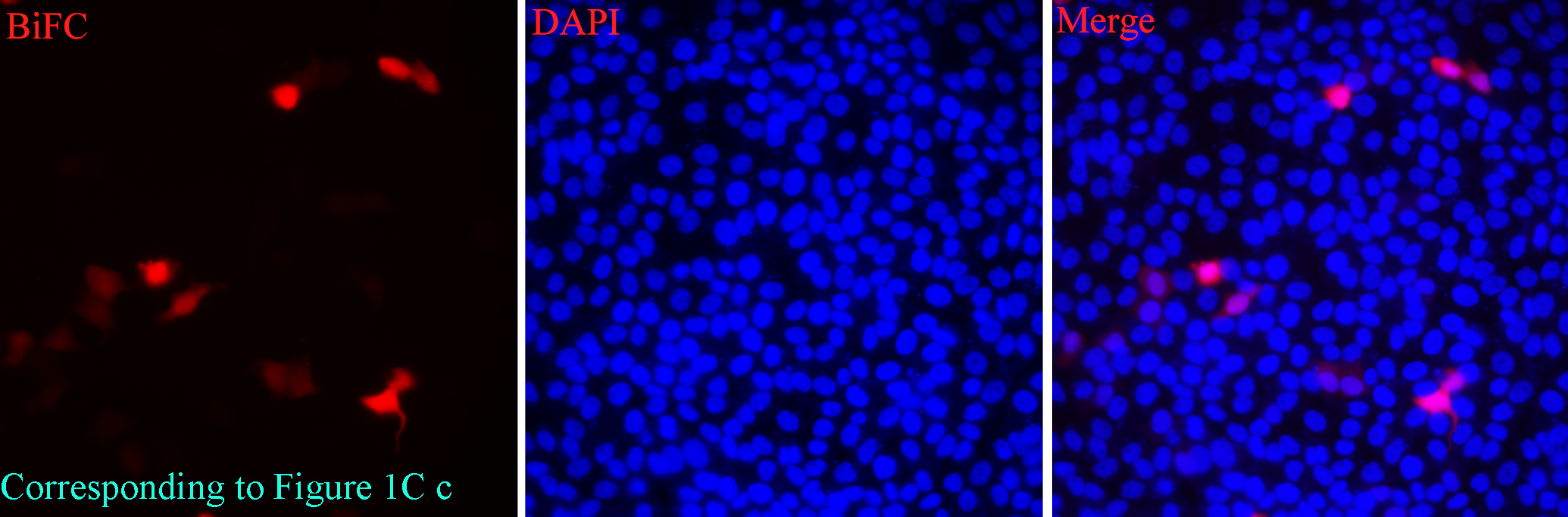

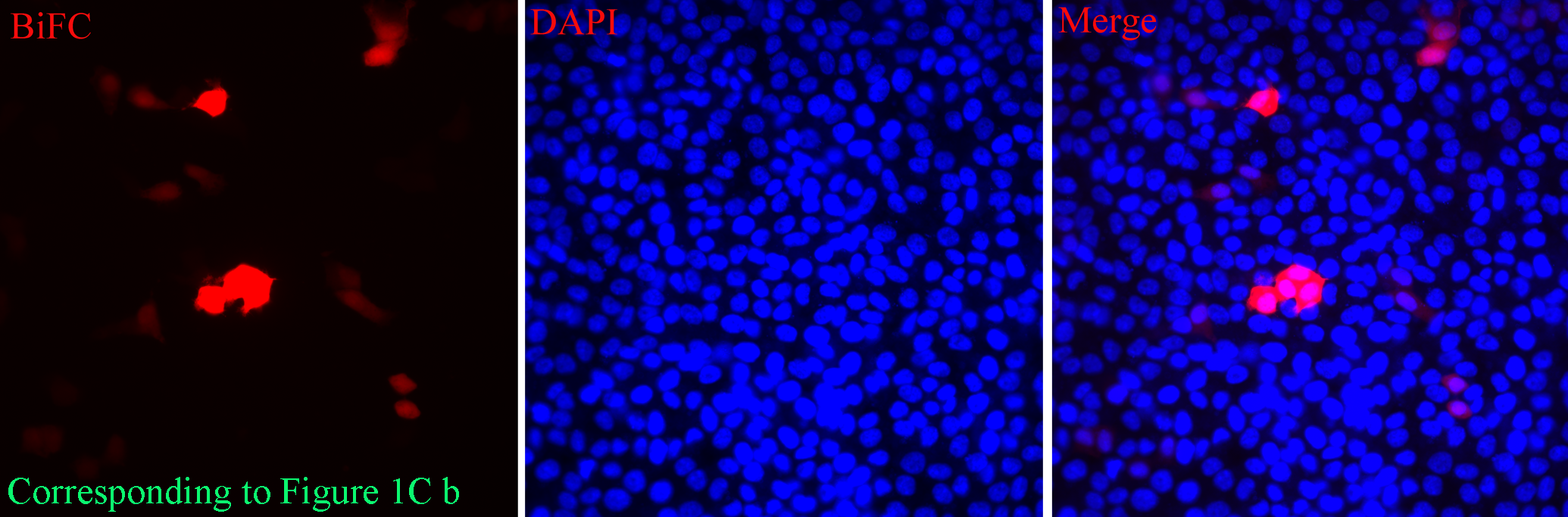

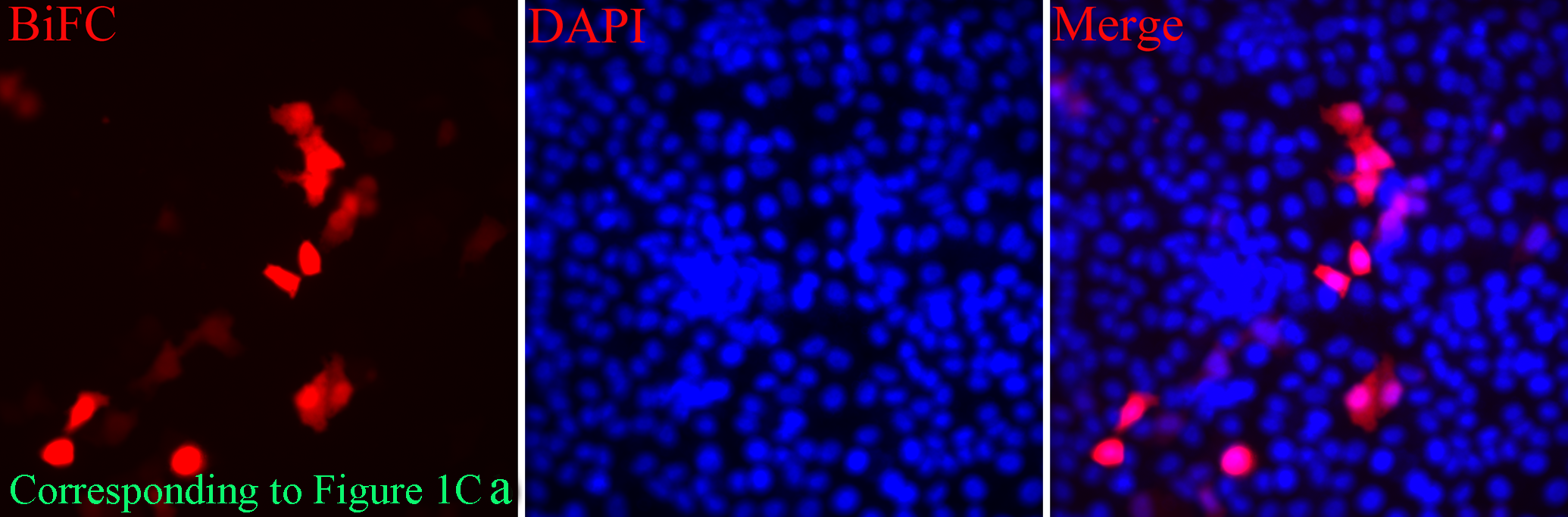

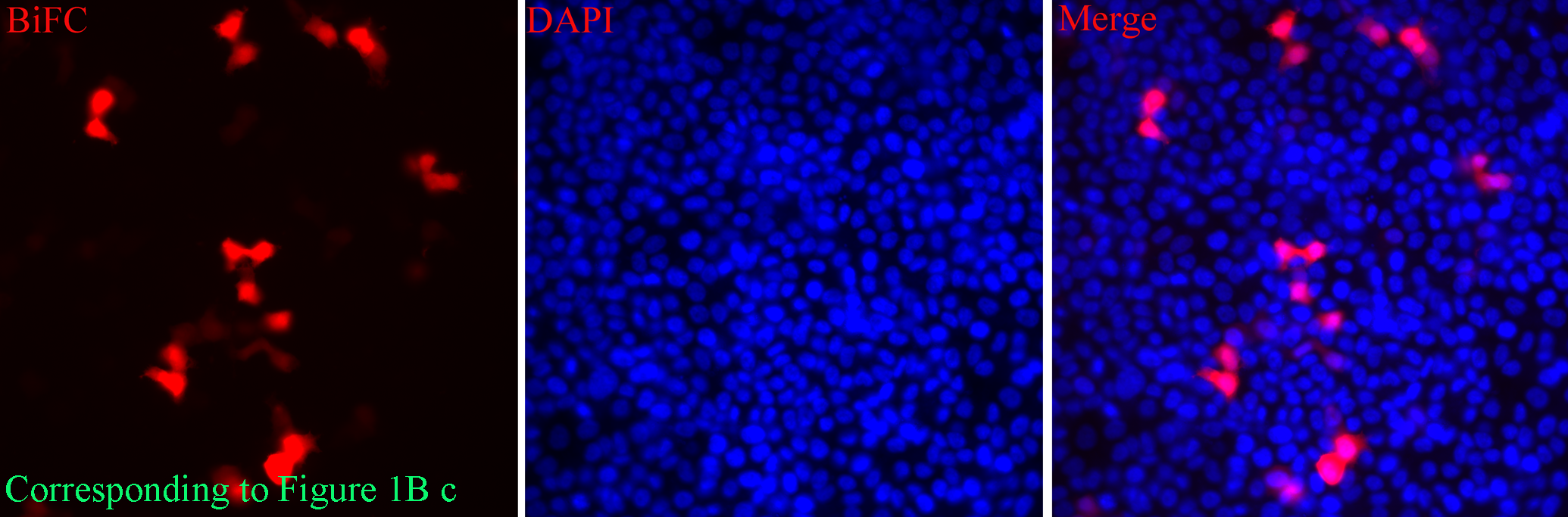


Reconstitution of RFP

untreated

Cotransfected with pcDNA-Myd88

Cotransfected with pcDNA-Hmgb1

6.7±0.3%

8.1±0.6%

1.8±0.2%**

1.6±0.2%**

**Figure S2.** **Reconstitution of RFP and the influence of HMGB1 and MyD88 on the Beclin1-Bcl2 heterodimer**. **(A)** Reconstitution of RFP. A549 cells were cotransfected with pMN-Bcl2 and pMC-Beclin1, after 8h, the cotransfected cells appeared a lot of red fluorescence. These graphs were corresponding to **Figure 1B c** in text. **(B)** The influence of HMGB1 and MyD88 on the Beclin1-Bcl2 heterodimer. Beclin1-binding proteins HMGB1 and MyD88 were expected to disrupt the Beclin1-Bcl2 heterodimer, after cotransfection for 8h, the cells were visualized, These graphs were corresponding to **Figure 1C a, b and c** in text. The ratios of RFP-positive cells were calculated in 5 fields chosen at random from three independent experiments, the data were shown as mean±SD, Data shown were the mean ± SD. * *P* < 0.05 and ** *P* < 0.01 vs. the untreated group.
