# Supplementary material for: Drug Screening for Autophagy Inhibitors Based on the Dissociation of Beclin1-Bcl2 Complex Using BiFC Technique and Mechanism of Eugenol on Anti-Influenza A Virus Activity
Source: PLoS One. 2013 Apr 16;8(4):e61026. doi: 10.1371/journal.pone.0061026 (PMC3628889; doi:10.1371/journal.pone.0061026)
Supplement: Figure S3 — The influence of ERK1/2 inhibitor (U0126, 10 µM), ERK1/2 activator (EGF, 100 ng/ml), JNK/p38 inhibitor (SB203580, 40 µM), p38 MAPK activator (anisomycin, 10 µM), antioxidant (NAC, 2 mM) and oxidant (H2O2, 100 µM) on the dissociation of Beclin1-Bcl2 heterodimer. After cotransfection, A549 cells were treated with these inhibitors and activators, after 8 h, the cells were visualized, These graphs were corresponding to Figure 1D a, b, c, d, e and f in text. The ratios of RFP-positive cells were calculated in 5 fields chosen at random from three independent experiments. Data shown were the mean ± SD. *P<0.05, **P<0.01. (DOC) [file pone.0061026.s003.doc]

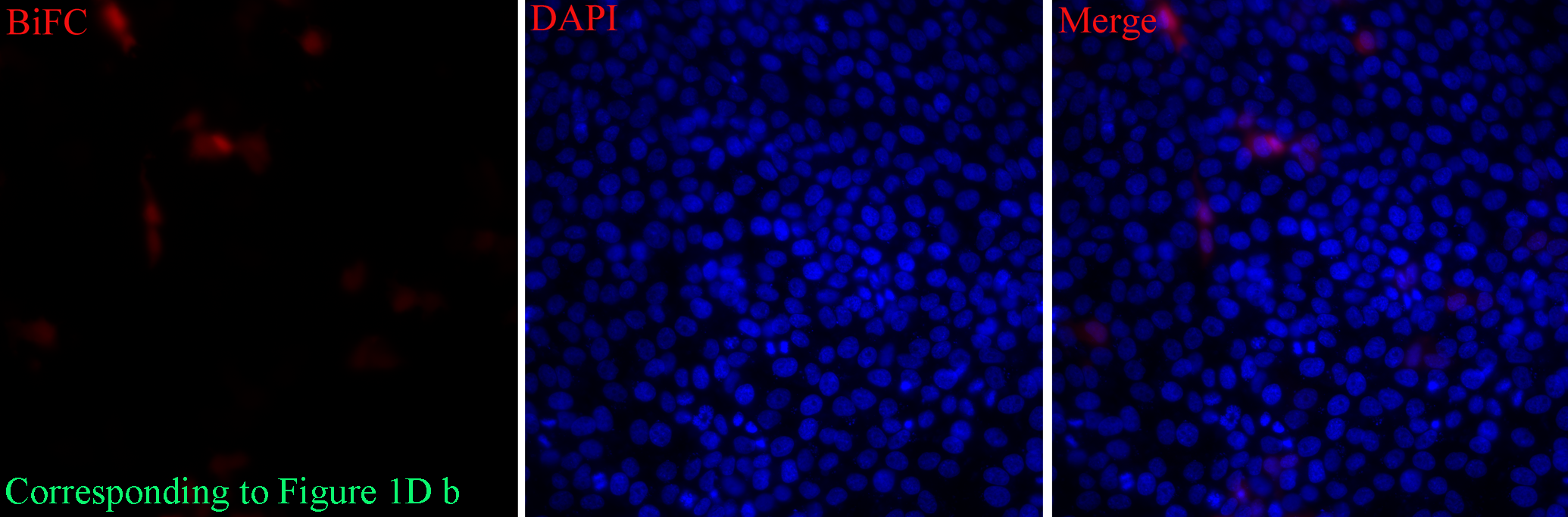

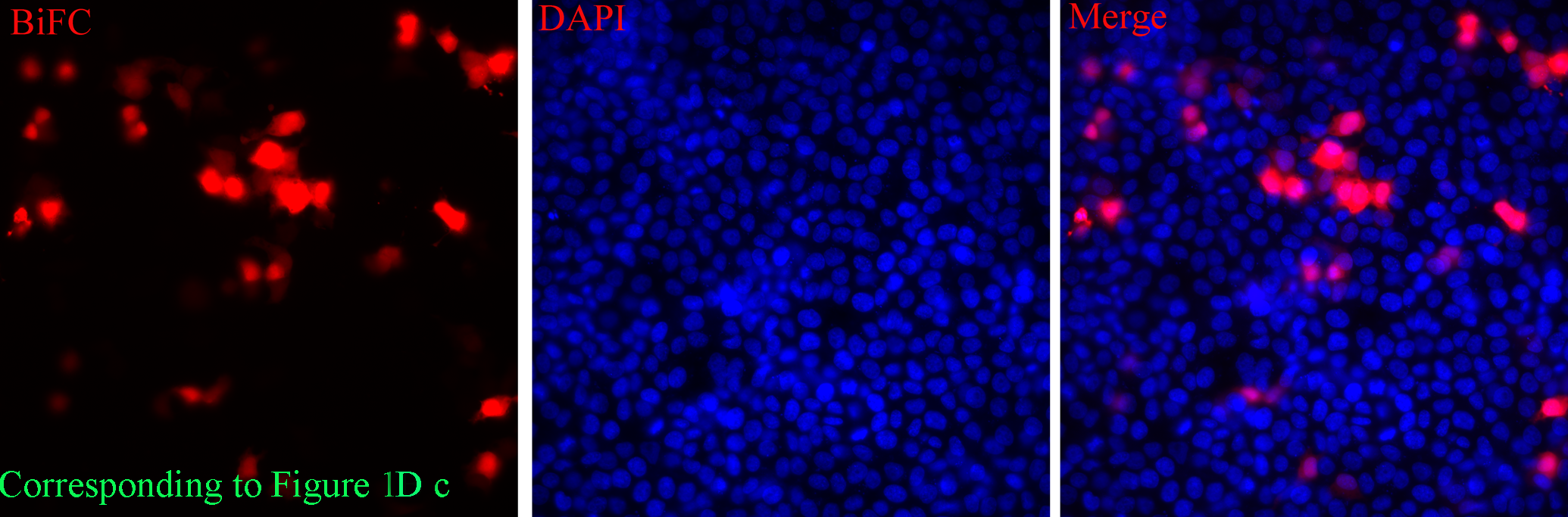

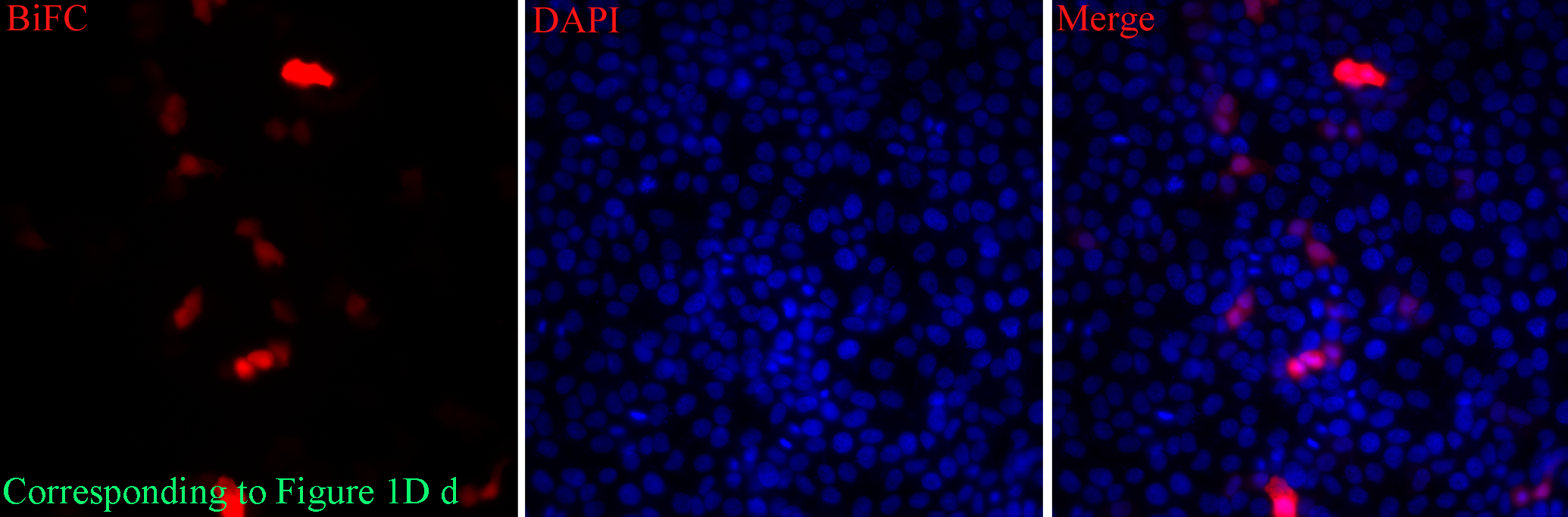

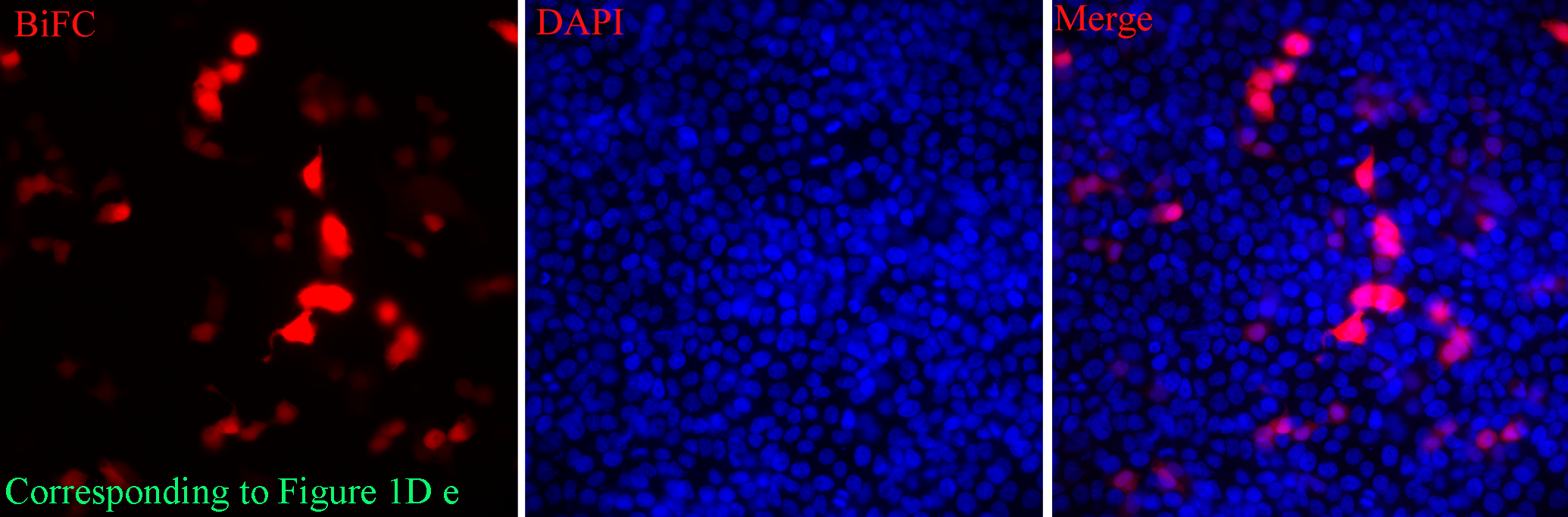

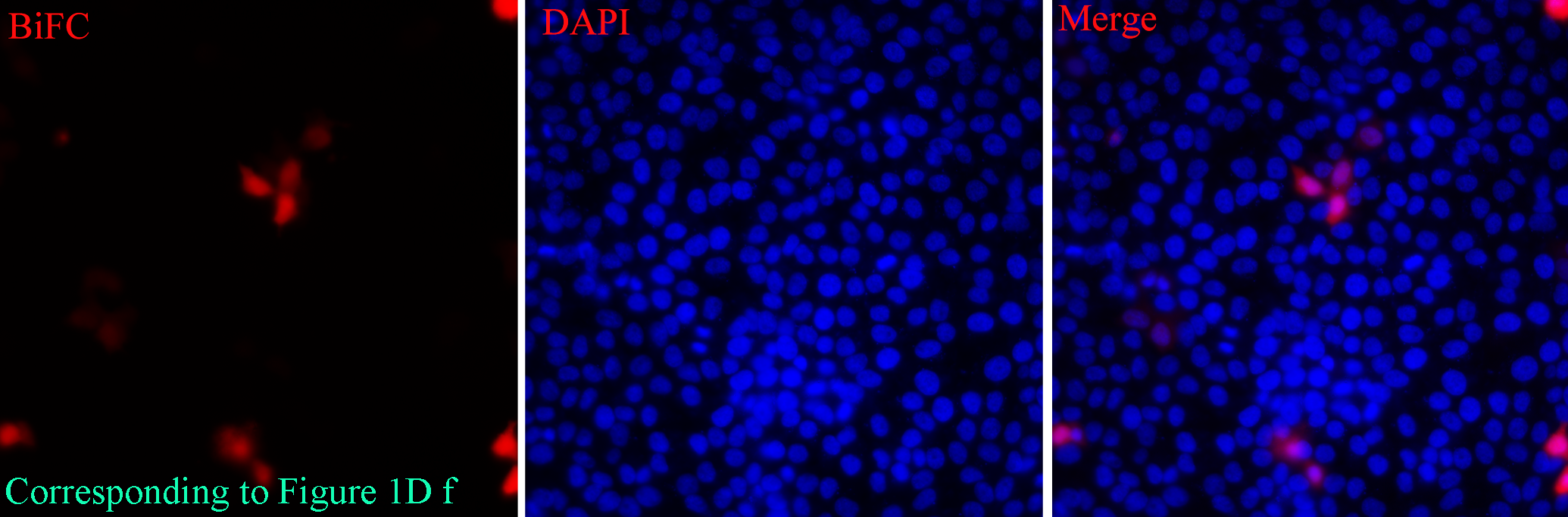

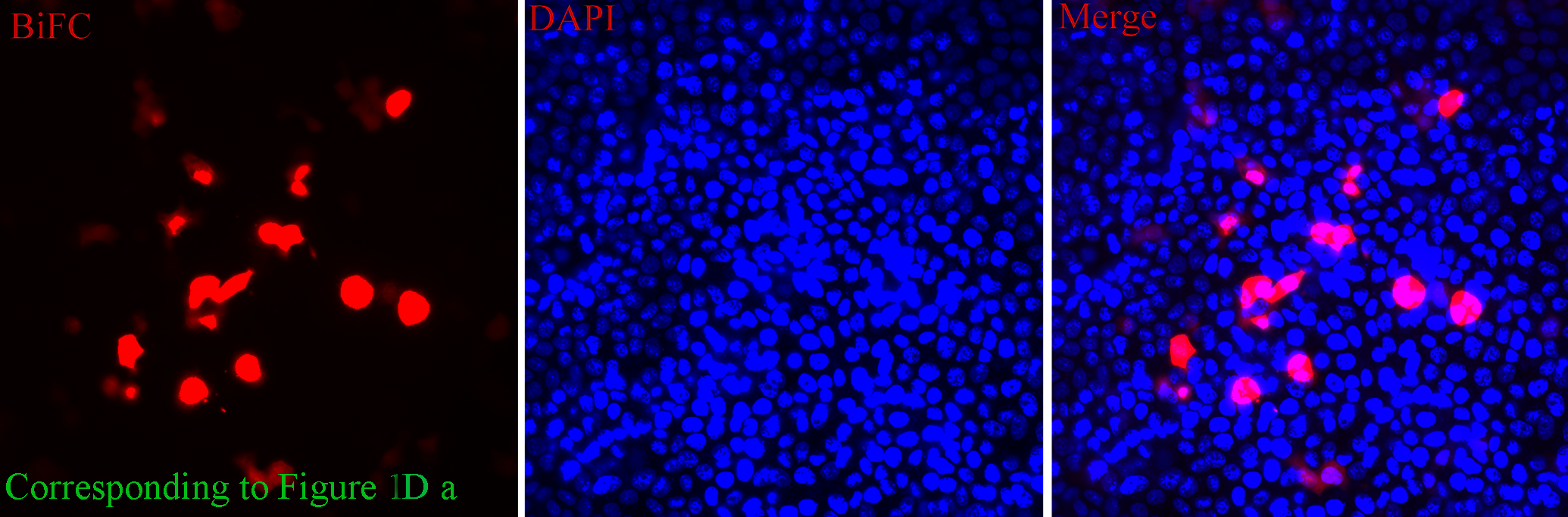


Treated with U0126

Treated with EGF

Treated with SB203580

Treated with Anisomycin

Treated with NAC

Treated with H_2_O_2_

2.0±0.2%**

7.7±0.6%

2.7±0.3%**

9.8±0.8%

1.0±0.2%**

5.5±0.6%

**Figure S3.** **The influence of ERK1/2 inhibitor (U0126, 10 μM), ERK1/2 activator (EGF, 100ng/ml), JNK/p38 inhibitor (SB203580, 40 μM), p38 MAPK activator (anisomycin, 10 μM), antioxidant (NAC, 2 mM) and oxidant (H2O2, 100 μM) on the dissociation of Beclin1-Bcl2 heterodimer**. After cotransfection, A549 cells were treated with these inhibitors and activators, after 8 h, the cells were visualized, These graphs were corresponding to **Figure 1D a, b, c, d, e** **and** **f** in text. The ratios of RFP-positive cells were calculated in 5 fields chosen at random from three independent experiments. Data shown were the mean ± SD. * *P* < 0.05, ** *P* < 0.01.
