# Supplementary material for: Drug Screening for Autophagy Inhibitors Based on the Dissociation of Beclin1-Bcl2 Complex Using BiFC Technique and Mechanism of Eugenol on Anti-Influenza A Virus Activity
Source: PLoS One. 2013 Apr 16;8(4):e61026. doi: 10.1371/journal.pone.0061026 (PMC3628889; doi:10.1371/journal.pone.0061026)
Supplement: Figure S4 — The influences of eugenol on the Beclin1-Bcl2 heterodimer with IAV infection. After cotransfection with pMC-Beclin1 and pMN-Bcl2, A549 cells were infected with IAV (A/ShanTou/169/06(H1N1)) (MOI = 2.0) and treated with ribavirin (25 µg/ml) and eugenol (5 µg/mL, after 8 h, the cells were visualized, these graphs were corresponding to Figure 2A a, b, c and d in text. The ratios of RFP-positive cells were calculated in 5 fields chosen at random from three independent experiments. Data shown were the mean ± SD. *P<0.05, **P<0.01 vs. the virus only group. (DOC) [file pone.0061026.s004.doc]

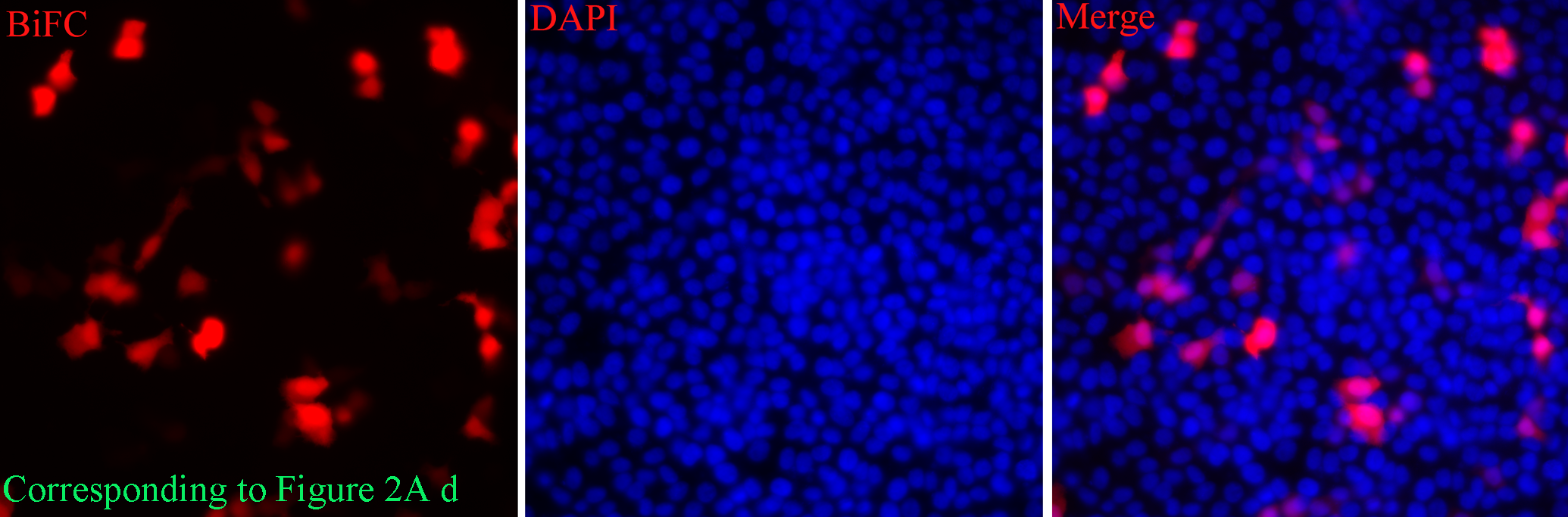

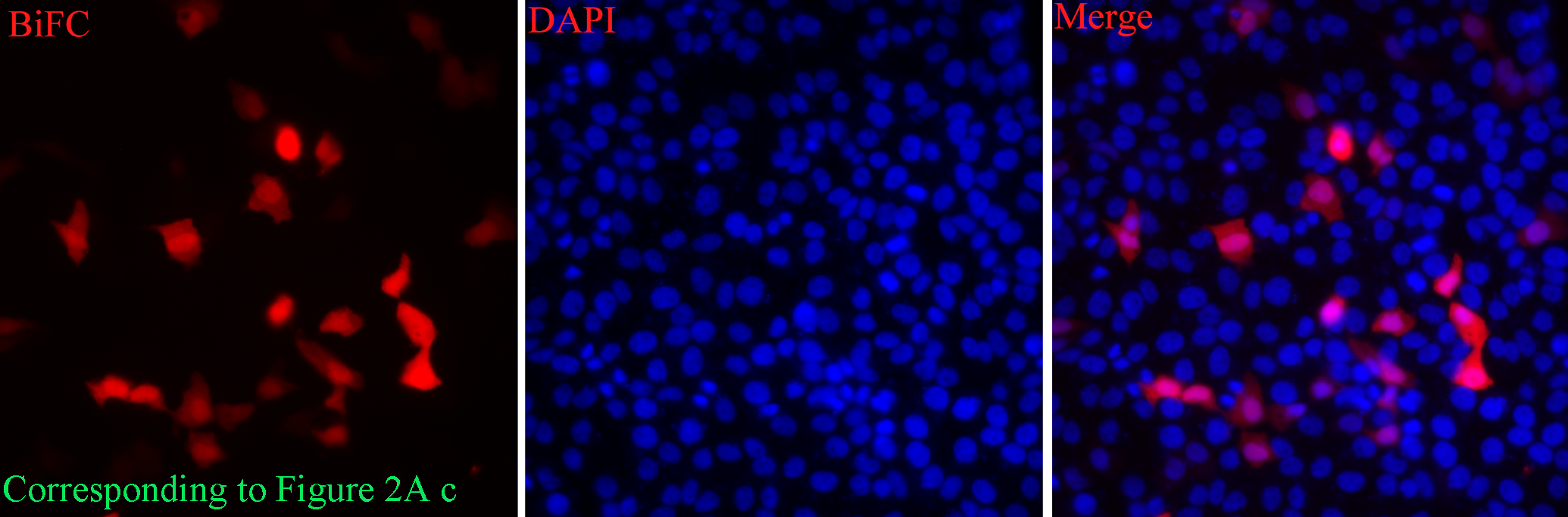

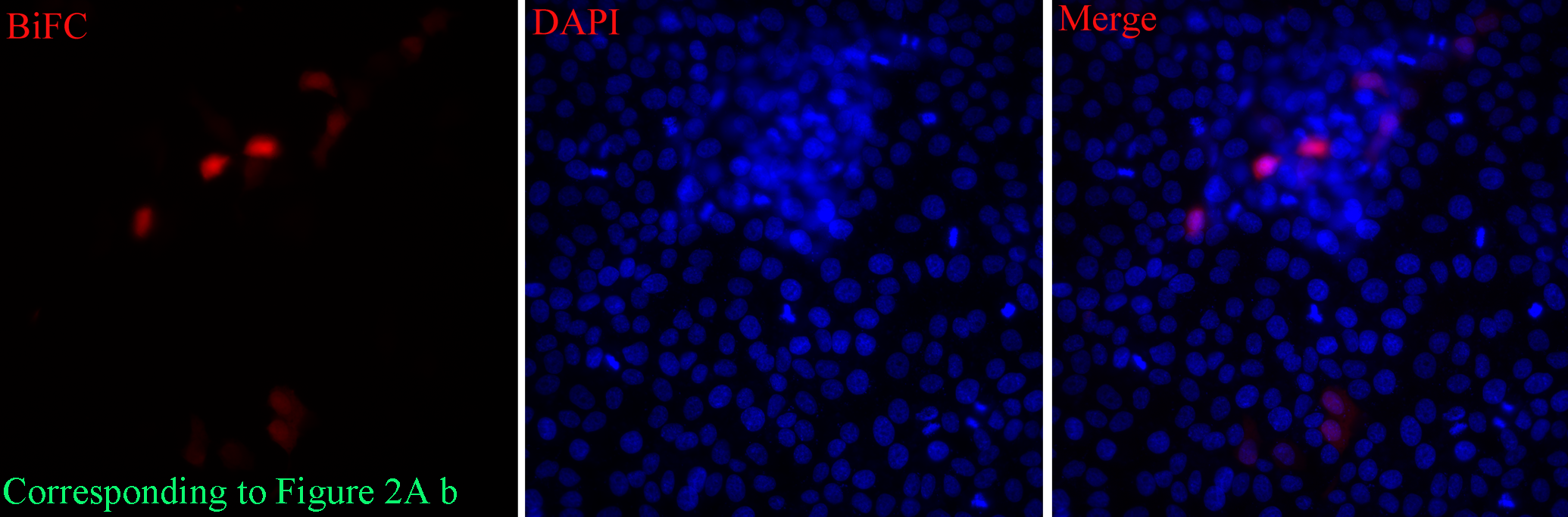

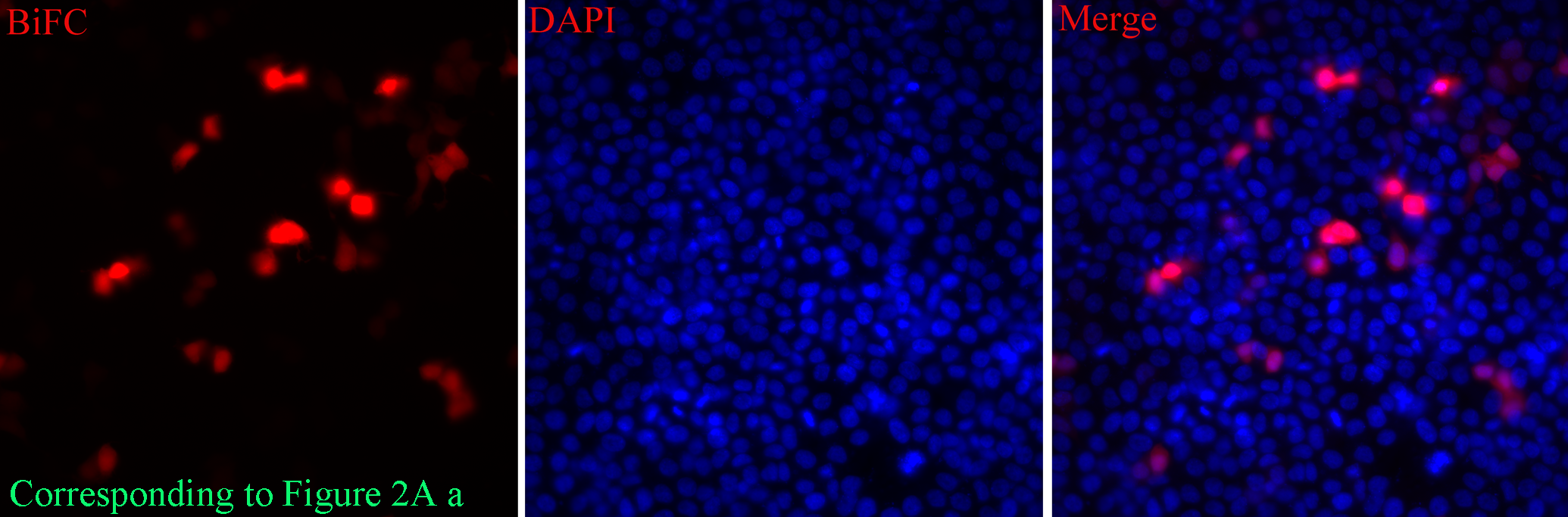


Untreated group

Virus only group

Ribavirin group

Eugenol group

7.7±0.7%**

1.4±0.2%

7.2±0.6%**

7.1±0.8%**

**Figure S4. The influences of eugenol on the Beclin1-Bcl2 heterodimer with IAV infection.** After cotransfection with pMC-Beclin1 and pMN-Bcl2, A549 cells were infected with IAV (A/ShanTou/169/06(H1N1)) (MOI = 2.0) and treated with ribavirin (25 μg/ml) and eugenol (5 μg/mL, after 8 h, the cells were visualized, these graphs were corresponding to **Figure 2A a, b, c and d** in text. The ratios of RFP-positive cells were calculated in 5 fields chosen at random from three independent experiments. Data shown were the mean ± SD. * *P* < 0.05, ** *P* < 0.01 vs. the virus only group.
