# Supplementary material for: Drug Screening for Autophagy Inhibitors Based on the Dissociation of Beclin1-Bcl2 Complex Using BiFC Technique and Mechanism of Eugenol on Anti-Influenza A Virus Activity
Source: PLoS One. 2013 Apr 16;8(4):e61026. doi: 10.1371/journal.pone.0061026 (PMC3628889; doi:10.1371/journal.pone.0061026)
Supplement: Figure S6 — Eugenol inhibited the accumulation of autophagosomes determined by EGFP-LC3 assay. A549 cells were transfected with the pEGFP-LC3 plasmid. In the untreated group, A549 cells were not infected with IAV. In the virus only treated group, A549 cells were infected but not treated with any drugs. In the ribavirin and Eug treated groups, A549 cells were infected and treated with ribavirin (25 µg/ml) and eugenol (5 µg/mL), respectively. The incubation time was 8 h, MOI = 2.0, The percentage of cells containing EGFP-LC3 dots to cells expressing EGFP was calculated in 10 fields chosen at random. The graphs were obtained from an inverted fluorescence microscope (10×40 and 10×100). Data shown were the mean ± SD of three independent experiments. *P<0.05 and **P<0.01 vs. NC. (DOC) [file pone.0061026.s006.doc]

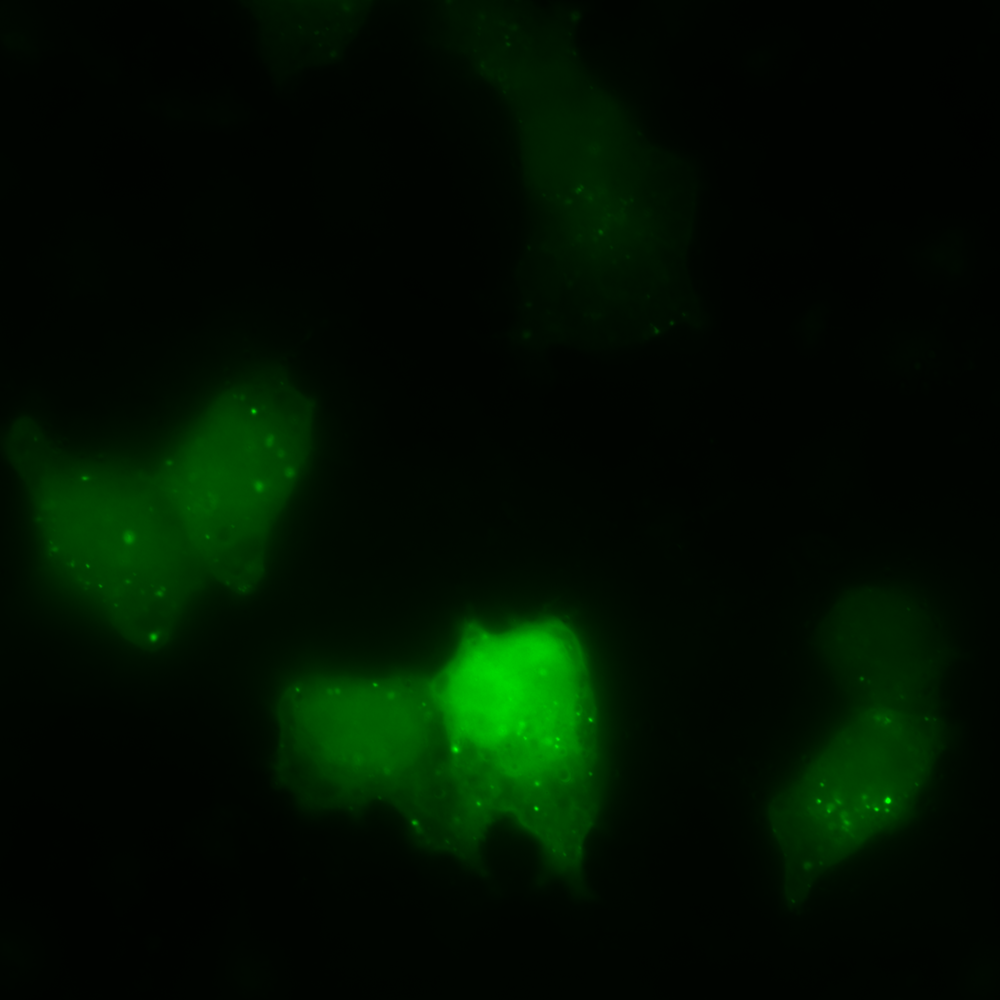

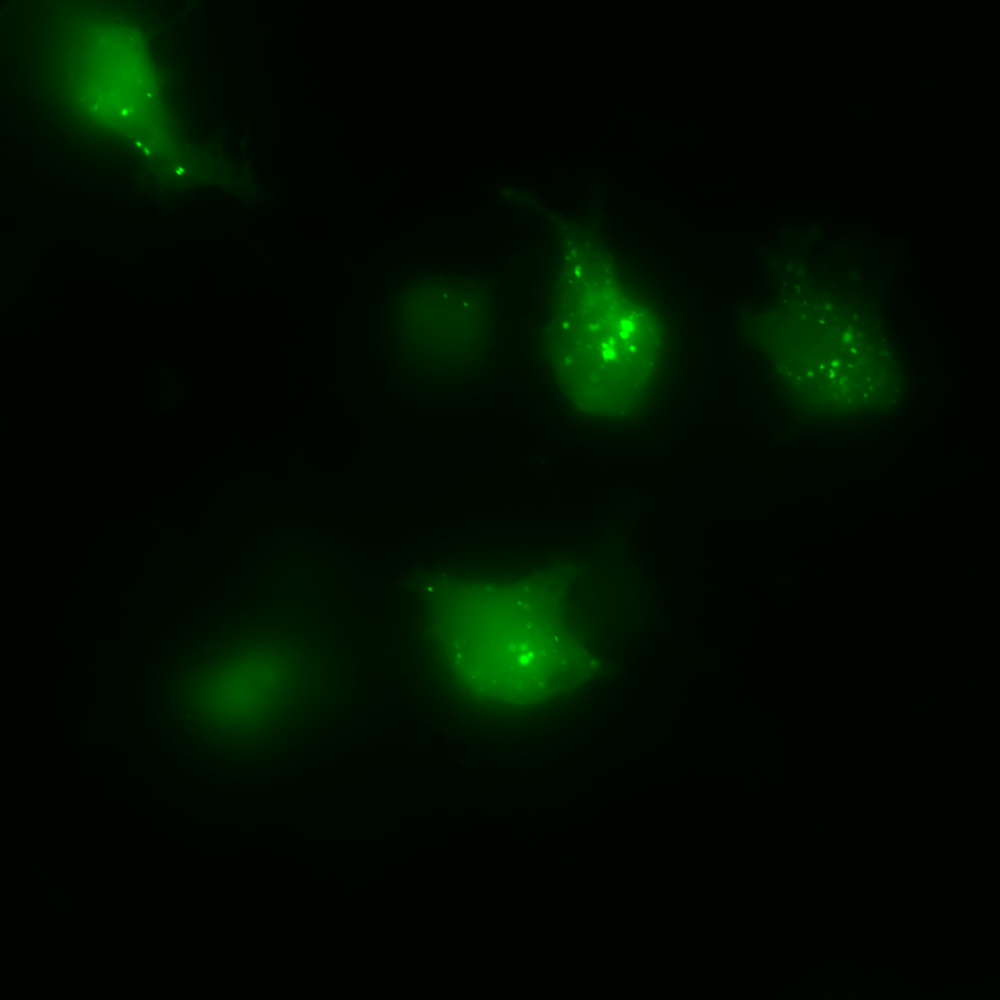

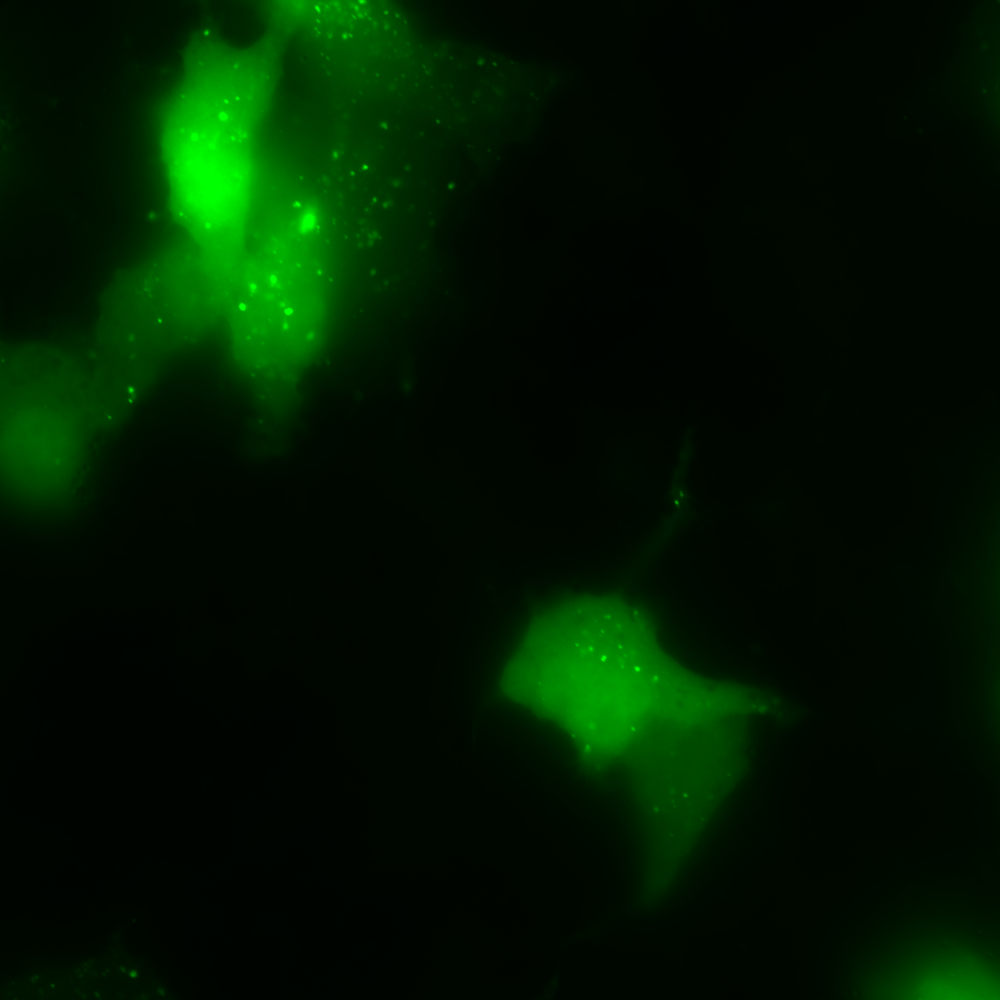

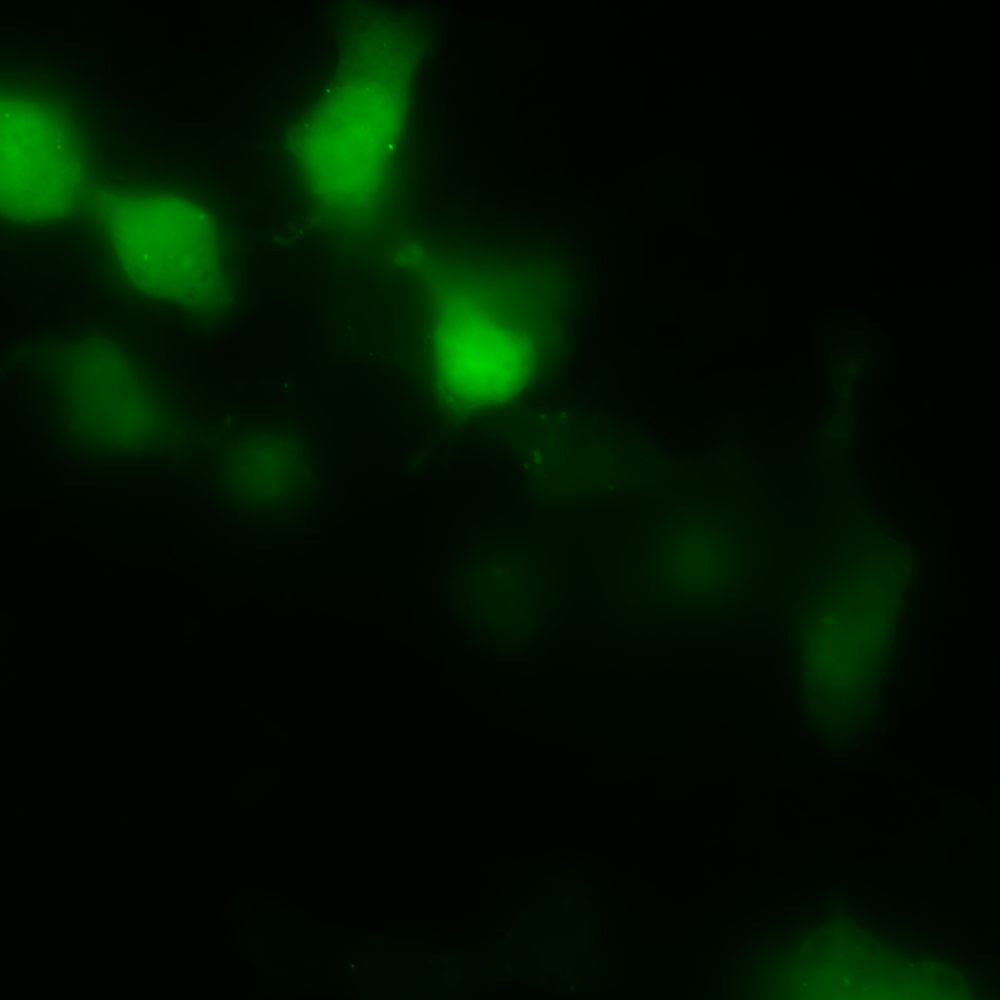

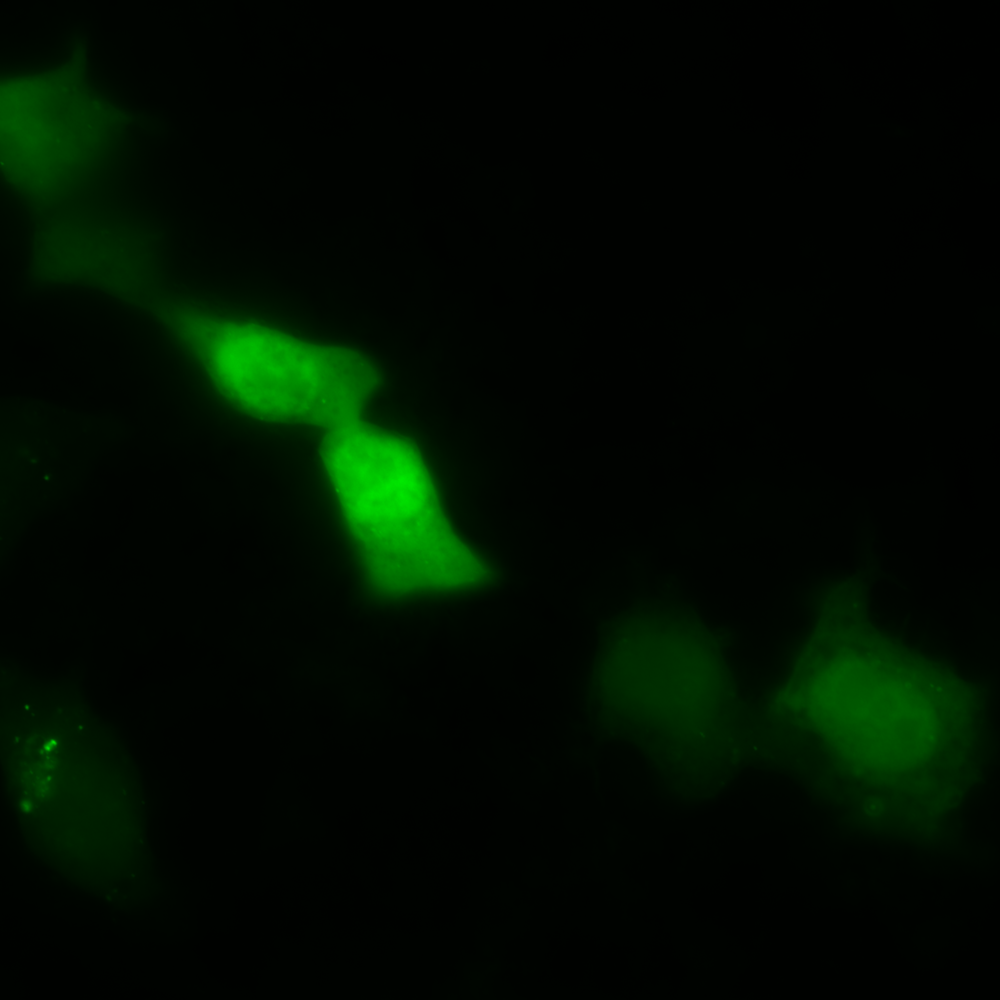

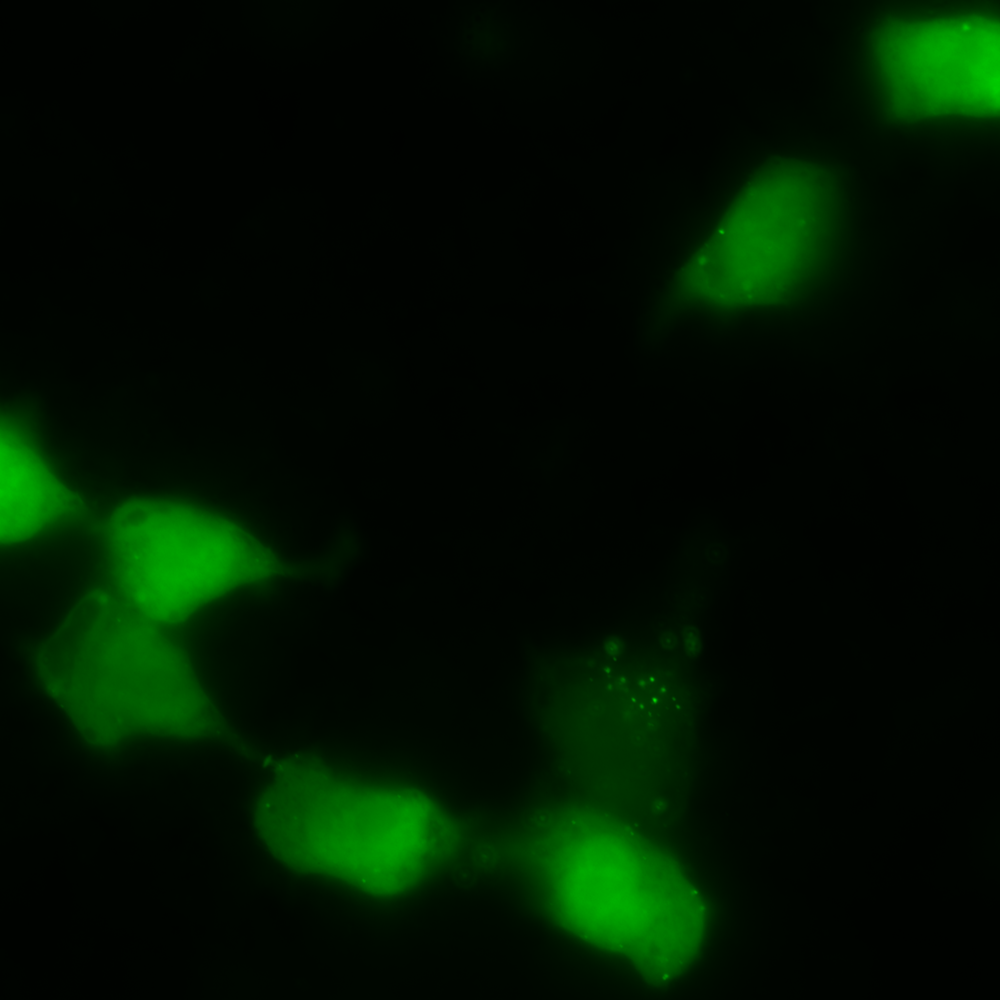

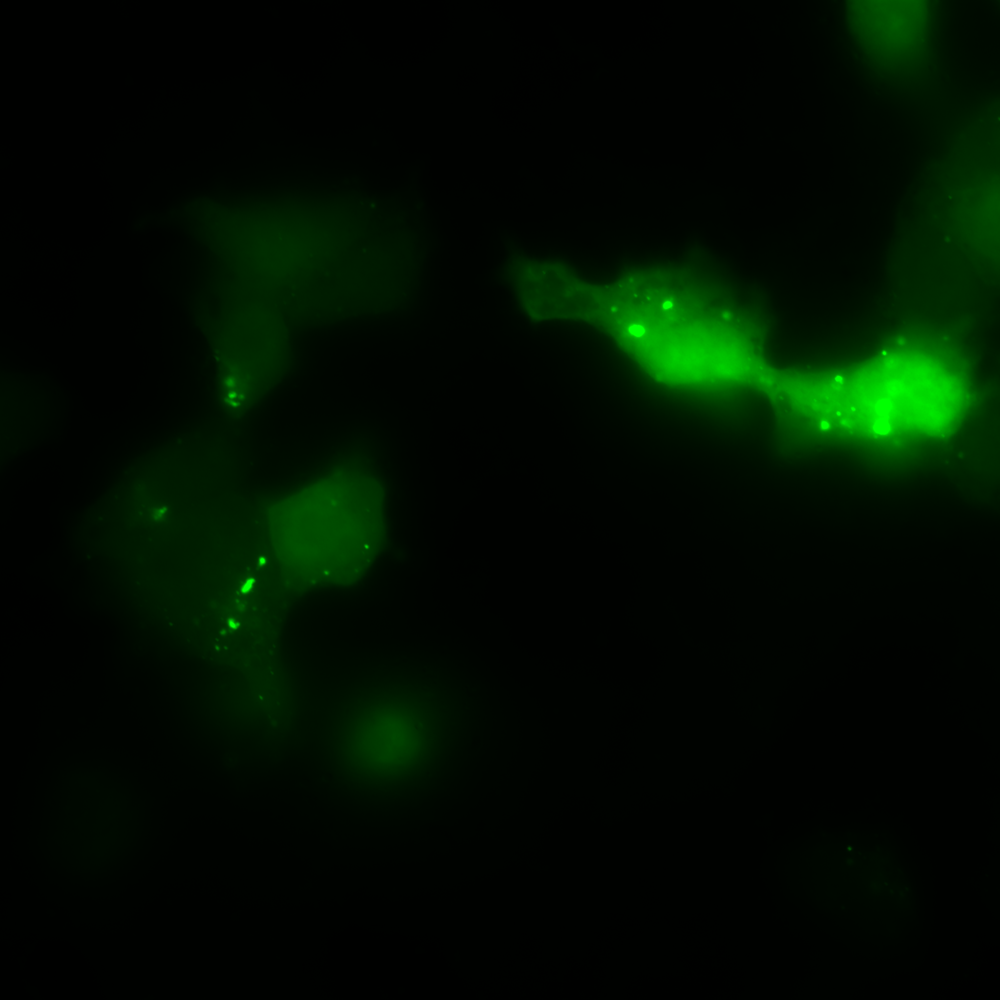

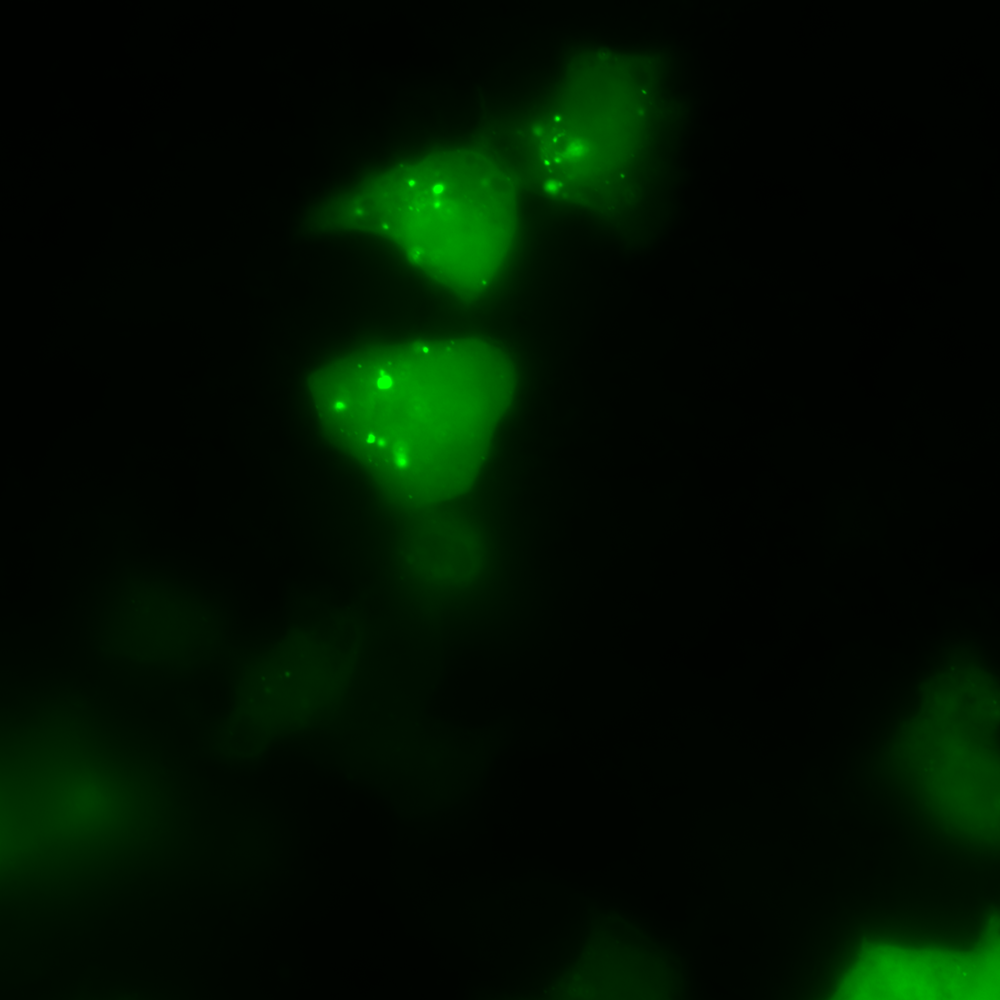

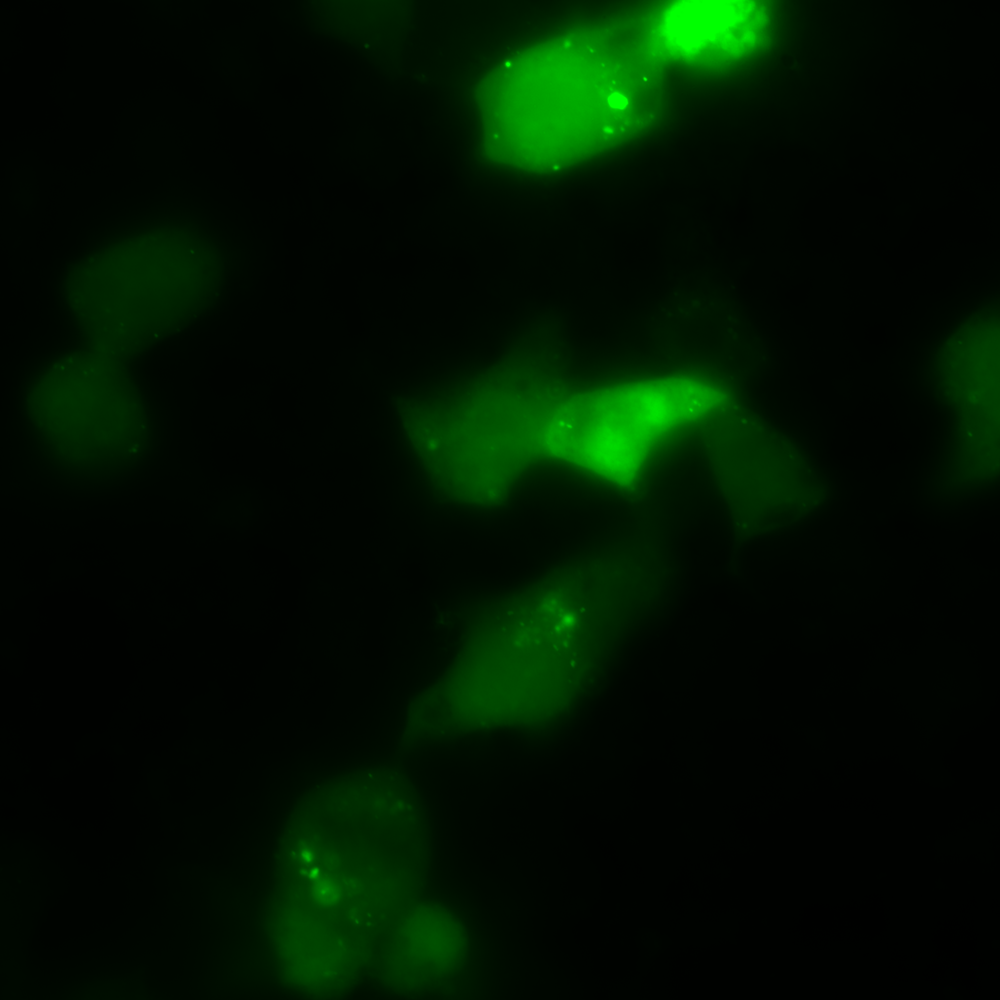

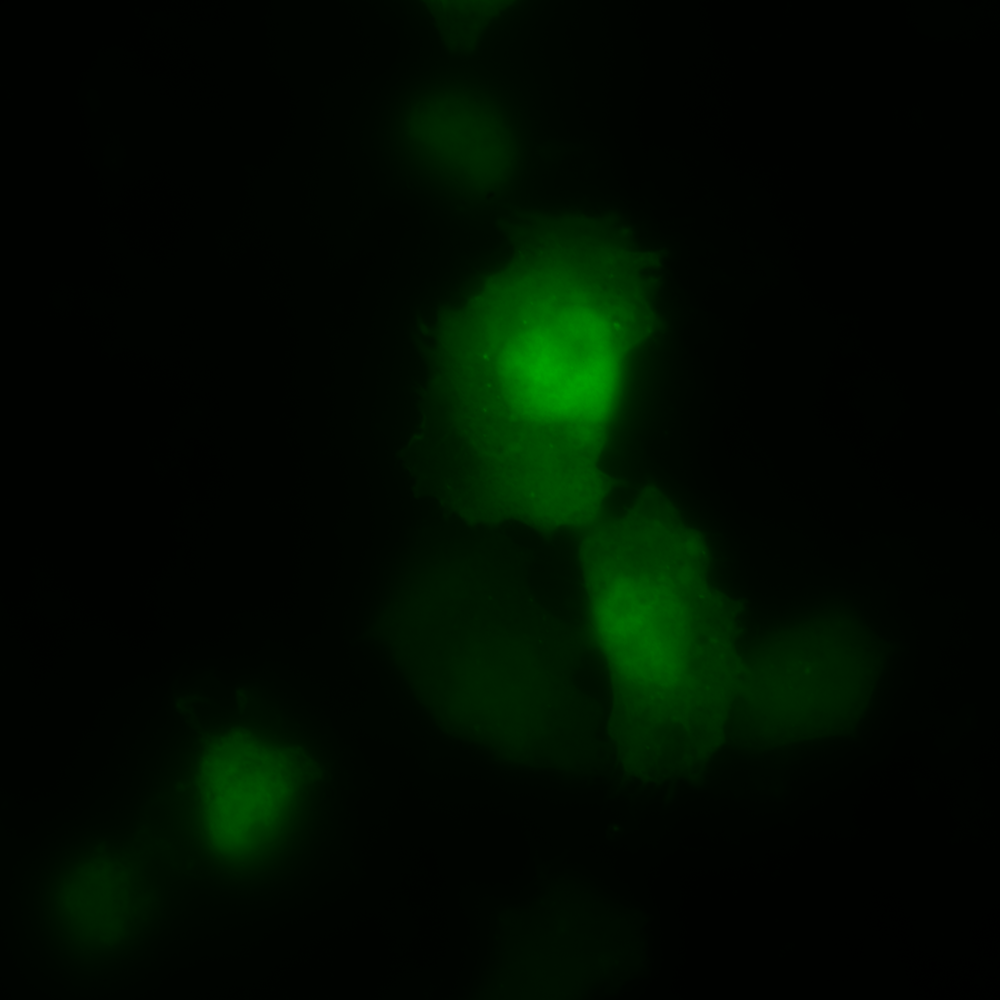

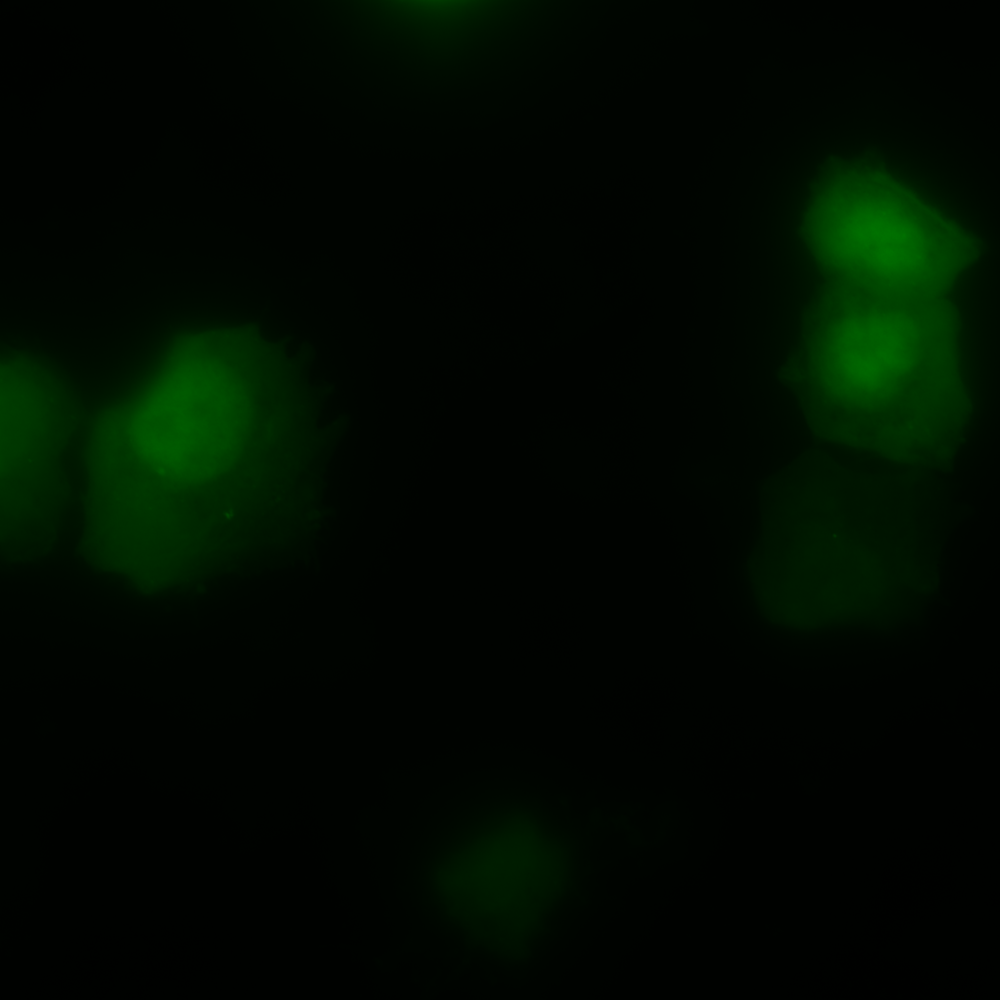

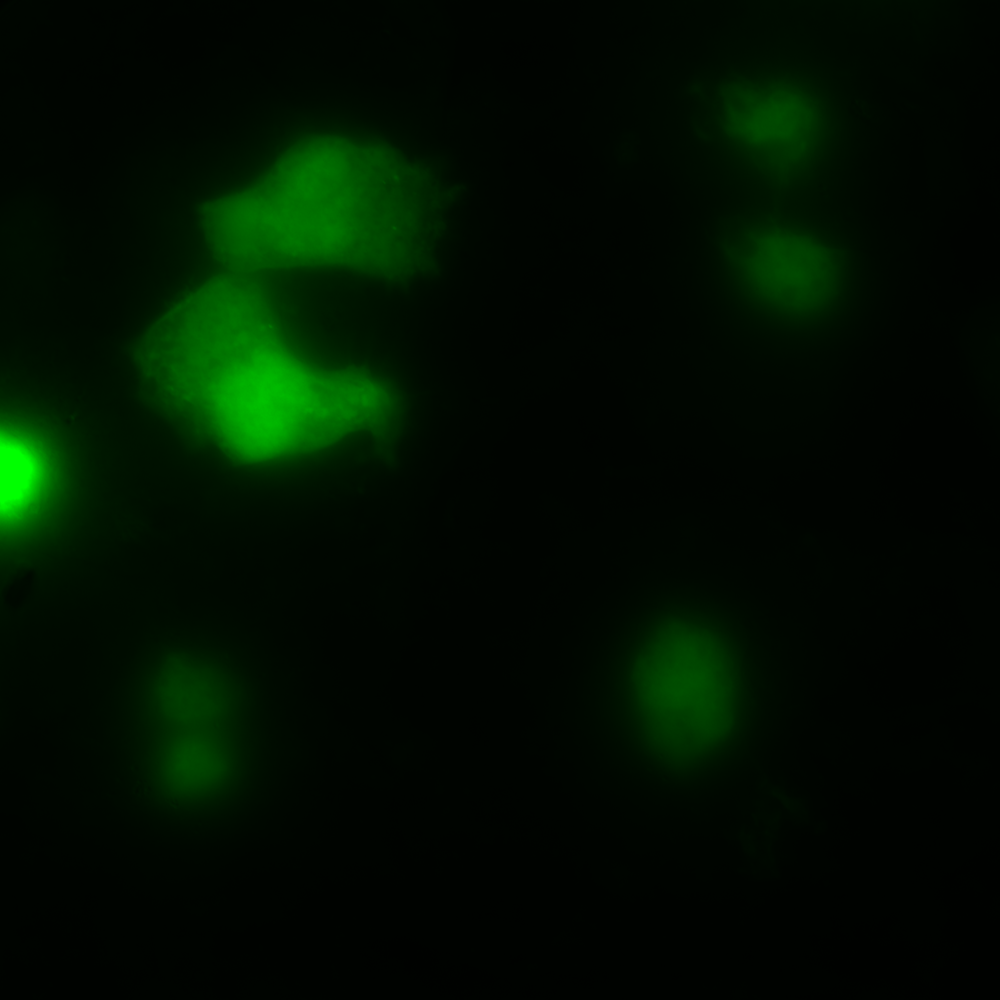


Untreated group (10×100)

Untreated group (10×100)

Untreated group (10×100)

Virus only group (10×100)

Virus only group (10×100)

Virus only group (10×100)

Ribavirin group (10×100)

Ribavirin group (10×100)

Ribavirin group (10×100)

Eugenol group (10×100)

Eugenol group (10×100)

Eugenol group (10×100)

**Figure S6**: **Eugenol inhibited the accumulation of autophagosomes determined by EGFP-LC3 assay**. A549 cells were transfected with the pEGFP-LC3 plasmid. In the untreated group, A549 cells were not infected with IAV. In the virus only treated group, A549 cells were infected but not treated with any drugs. In the ribavirin and Eug treated groups, A549 cells were infected and treated with ribavirin (25 μg/ml) and eugenol (5 μg/mL), respectively. The incubation time was 8 h, MOI = 2.0, The percentage of cells containing EGFP-LC3 dots to cells expressing EGFP was calculated in 10 fields chosen at random. The graphs were obtained from an inverted fluorescence microscope (10×40 and 10×100). Data shown were the mean ± SD of three independent experiments. * *P* < 0.05 and ** *P* < 0.01 vs. NC.
