# Supplementary material for: Drug Screening for Autophagy Inhibitors Based on the Dissociation of Beclin1-Bcl2 Complex Using BiFC Technique and Mechanism of Eugenol on Anti-Influenza A Virus Activity
Source: PLoS One. 2013 Apr 16;8(4):e61026. doi: 10.1371/journal.pone.0061026 (PMC3628889; doi:10.1371/journal.pone.0061026)
Supplement: Figure S7 — Rapamycin antagonized the anti-IAV activity of eugenol. A549 cells were infected (MOI = 0.001) and treated with eugenol (5 µg/mL), and simultaneously treated with or without various concentrations of rapamycin (0, 0.625, 1.25, 2.5, 5, 10 µM), after 48 h, the antiviral activity (cell viability) was determined by the SRB method using CPE reduction (A). In another experiment, the supernatants were collected and the virus titers were determined by a plaque assay on MDCK cells (B). Additionally, rapamycin could promote the replication of IAV (C). Data shown were the mean ± SD of three independent experiments. *p<0.05, **p<0.01.vs the virus+eugenol. (DOC) [file pone.0061026.s007.doc]

Antiviral activity

(% of the control)

Concentration of rapamycin (μM)

**A**


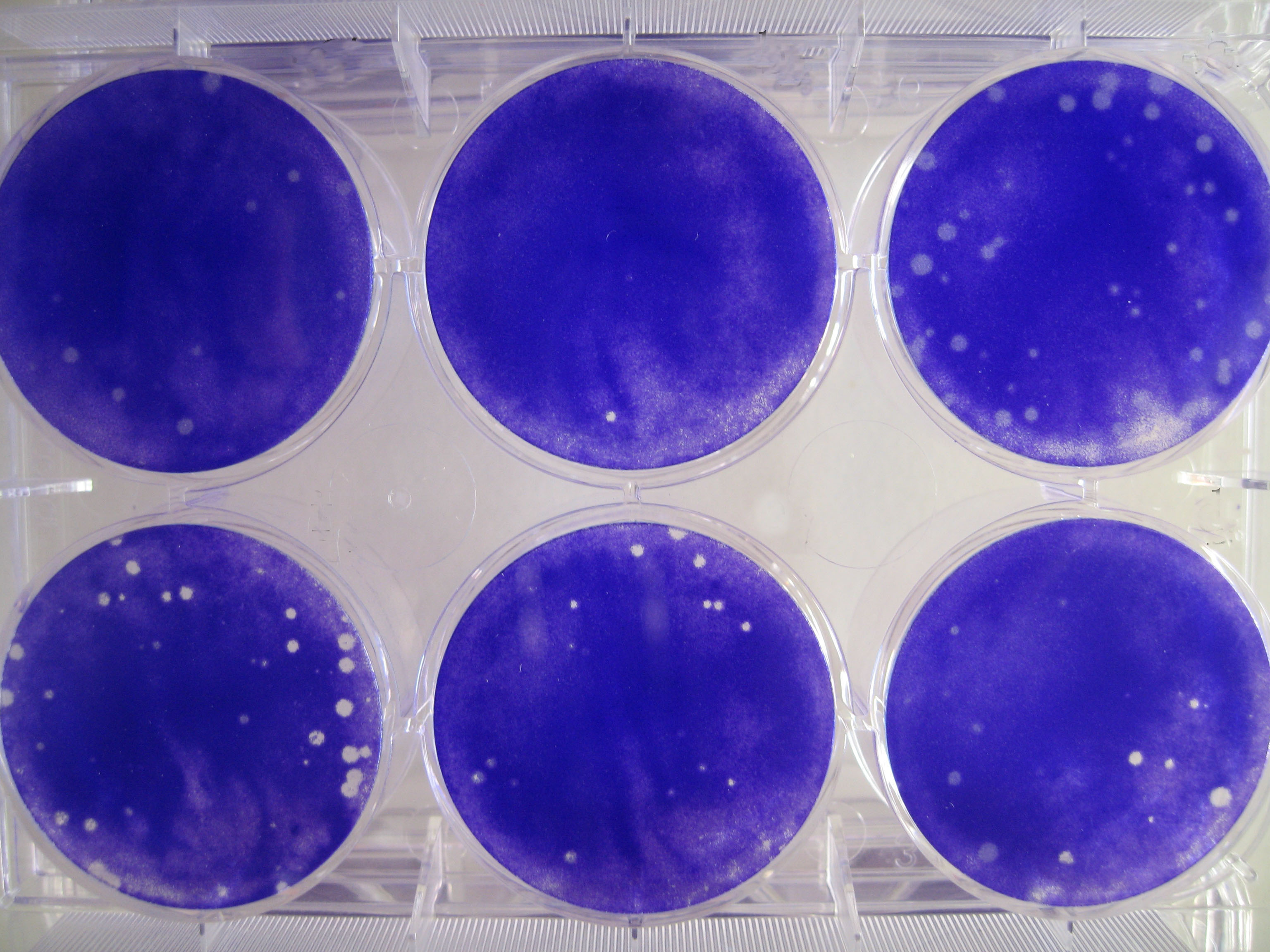


**B**

Virus only

Virus +

eugenol (5μg/mL)

Virus +

eugenol (5μg/mL) +

rapamycin (0.625μM)

Virus +

eugenol (5μg/mL) +

rapamycin (1.25μM)

Virus +

eugenol (5μg/mL) +

rapamycin (2.5μM)

Virus +

eugenol (5μg/mL) +

rapamycin (5μM)


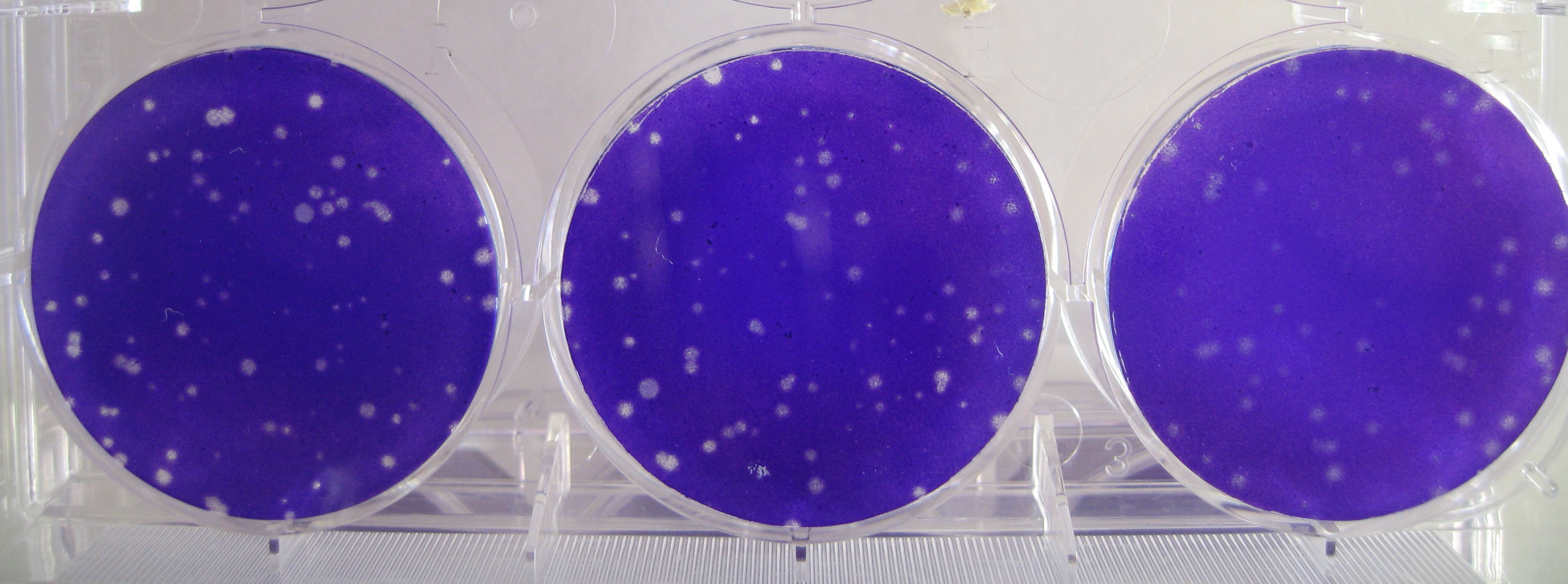


**C**

Virus only

Virus +

rapamycin (5 μM)

Virus +

rapamycin (10 μM)

**Figure S7. Rapamycin antagonized the anti-IAV activity of eugenol.** A549 cells were infected (MOI = 0.001) and treated with eugenol (5μg/mL), and simultaneously treated with or without various concentrations of rapamycin (0, 0.625, 1.25, 2.5, 5, 10 μM), after 48 h, the antiviral activity (cell viability) was determined by the SRB method using CPE reduction (**A**). In another experiment, the supernatants were collected and the virus titers were determined by a plaque assay on MDCK cells (**B**). Additionally, rapamycin could promote the replication of IAV (**C**). Data shown were the mean ± SD of three independent experiments. * *p* < 0.05, ** *p* < 0.01.*vs* the virus + eugenol.
