# Supplementary material for: Drug Screening for Autophagy Inhibitors Based on the Dissociation of Beclin1-Bcl2 Complex Using BiFC Technique and Mechanism of Eugenol on Anti-Influenza A Virus Activity
Source: PLoS One. 2013 Apr 16;8(4):e61026. doi: 10.1371/journal.pone.0061026 (PMC3628889; doi:10.1371/journal.pone.0061026)
Supplement: Table S1 — Influence of plant extracts on the dissociation of the Beclin1-Bcl2 heterodimer (fold change). A549 cells were seeded in 96-well plate for 24 h, then cotransfected with pMC-Beclin1 and pMN-Bcl2 plasmids, after 6 h, in the blank group (BG, untreated), A549 cells were not infected with IAV; in the negative control (NC, virus only) group, A549 cells were infected, but not treated with any drugs; in the positive control (PC, ribavirin) and test drug groups, A549 cells were infected, and treated with ribavirin (25 µg/ml) and test drugs at the maximum no-cytotoxicity concentrations, respectively. MOI = 2.0, the incubation times were 8 h. The FI was determined at 610 nm after excitation at 587 nm using a microplate reader (Tecan infinite M1000). All numbers were expressed as fold change relative to the negative control (NC, virus only). Data shown were mean ± SD from three independent experiments performed in triplicate.*P<0.05, **P<0.01 vs. the negative control (NC, virus only). (DOC) [file pone.0061026.s008.doc]

**Table S1. Influence of plant extracts on the dissociation of the Beclin1-Bcl2 heterodimer (fold change)**

| Treatment group | mean ± SD | Treatment group | mean ± SD |
| --- | --- | --- | --- |
| BG (untreated) | 2.78±0.29** | NC (virus only) | 1.00±0.00 |
| PC (ribavirin) | 1.49±0.12* | *Arctostaphylos uvaursi* subsp. cratericola | 1.07±0.25 |
| *Hibiscus mutabilis* L. | 1.35±0.30 | *Cyperus rotundus* L. | 1.11±0.19 |
| *Stemona sessilifolia* (Miq.) Miq. | 1.36±0.39 | *Salvia miltiorrhiza* Bge. | 1.11±0.19 |
| *Astragalus membranaceus* (Fisch.) Bge. | 1.37±0.40 | *Radix Semiaquilegiae* | 1.22±0.15 |
| *Genista tinctoria* Linn. | 1.37±0.42 | *Bambusa tuldoides* Munro. | 1.24±0.20 |
| *Sophora subprostrala* Chun et T.Chen. | 1.32±0.09 | *Glechoma hederacea* L. | 1.23±0.26 |
| *Arctium lappa* L. | 1.45±0.15* | *Rohdea japonica* Roth. | 1.25±0.36 |
| *Mentha haplocalyx* Briq. | 1.40±0.47 | *Euphorbia hirta.* L. | 1.38±0.11* |
| *Saposhnikovia divaricata* (Turcz.) Schischk. | 1.39±0.47 | *Xanthium sibiricun* Patr. | 1.30±0.27 |
| *Artemisia argyi* Levl. et Vant. | 1.39±0.45 | *Bos taurus domesticus* Gmelin. | 1.13±0.25 |
| *Kaempferia galanga* L. | 1.27±0.10 | *Bolbostemma paniculatum* Franquet. | 1.06±0.23 |
| *Pogostemon cablin* (Blanco) Benth. | 1.50±0.20* | *Trichosanthes Kirilowii* Maxim. | 1.05±0.19 |
| *Citrus reticulate* Blanco. | 1.22±0.01 | *Vitex trifolia* L. | 1.22±0.20 |
| *Paeonia lactiflora* Pall. | 1.37±0.40 | *Equisetum hiemale* L. | 1.24±0.24 |
| *Indigo naturalis* | 1.35±0.37 | *Erodium stephanianum* Willd. | 1.11±0.23 |
| *Andrographis paniculata* (Burm. F. ) Nees. | 1.21±0.01 | *Siegesbeckia orientalis* L. | 1.12±0.21 |
| *Pogostemon cablin* (Blanco) Benth. | 1.37±0.40 | *Punica granatum* L. | 1.56±0.17** |
| *Anglica dahurica* Benth.ex Hook | 1.40±0.47 | *Eucommia ulmoides* Oliv. | 1.29±0.17 |
| *Smilax glabra Roxb*. | 1.36±0.38 | *Pulsatilla chinensis* (Bge.)Reg. | 1.24±0.26 |
| *Scutellaria barbata* D. Don. | 1.39±0.47 | *Fraxinus rhynchophylla* Hance. | 1.22±0.31 |
| *Glycyrrhiza uralensis* Fisch. | 1.70±0.13** | *Glycine max* (L.)Merr. | 1.22±0.10 |
| *Polygonum cuspidatum* Sieb. et Zucc. | 1.35±0.36 | *Polygonatum sibiricum* Red. | 1.22±0.13 |
| *Curcuma phaeocaulis* Val. | 1.40±0.47 | *Pinus tabulaefomis* Carr. | 1.21±0.32 |
| *Mahonia fortunei* (Lindl.)Fedde | 1.26±0.11 | *Chaenomeles speciosa* (Sweet) Nakai | 1.09±0.24 |
| *Morus alba* L. | 1.25±0.06 | *Achyranthes bidentata* Bl. | 1.10±0.19 |
| *Aristolochia debilis* Seib.et Zucc. | 1.37±0.39 | *Platycladus orientalis* (L.) Franco. | 1.16±0.10 |
| *Reseda odorata* L. | 1.36±0.25 | *Panax notoginseng* (Burt.) F. H. Chen. | 1.12±0.22 |
| *Kaempferia galanga* L. | 1.57±0.15* | *Rbia cordifolia* L. | 1.17±0.11 |
| *Acacia catechu* (L.F.) Willd. | 1.46±0.15* | *Sanhuisorba officinalis* L. | 1.16±0.15 |
| *Lantana camara* L. | 1.30±0.19 | *Hypericum japonicum* Thunb. | 1.16±0.24 |
| *Sophora flavescens* Ait. | 1.45±0.10* | *Patrinia scabiosaefolia* Fisch. | 1.06±0.13 |
| *Verbena officinalis* L. | 1.29±0.13 | *Dryopteris setosa* (Thunb.) Akasawa | 1.17±0.17 |
| *Serissa serissoides* (DC.) Druce | 1.30±0.15 | *Aloe barbadensis* Miller. | 1.03±0.20 |
| *Peucedanum praeruptorum* Dunn. | 1.50±0.11* | *Ginkgo biloba* L. | 1.39±0.12 |
| *Borneolum syntheticum* | 1.56±0.10* | *Hedyotis diffusa* Willd. | 0.99±0.20 |
| *Schizonepeta tenuifolia* Briq. | 1.34±0.28 | *Litsea cubeba* (Lour.) Pers. | 1.08±0.16 |
| *Rheum palmatum* L. | 1.31±0.22 | *Apium graveolens* L. var. dulce DC. | 1.58±0.11** |
| *Kummerowia striata* (Thunb.) Schindl | 1.27±0.11 | *Scutellaria baicalensis* Georgi. | 1.20±0.12 |
| *Chrysanthemum indicum* L. | 1.28±0.12 | ***Syzygium aromaticum* L.** | 1.88±0.32** |
| *Coptis chinensis* Franch. | 1.55±0.18* | *Evodia rutaecarpa* (Juss.) Benth. | 1.34±0.08 |
| *Areca catechu* L. | 1.39±0.10 | *Curcuma longa* L. | 1.62±0.10** |
| *Buddleja lindleyana* Fort. | 1.65±0.16** | *Cinnamomum cassia* Presl | 1.43±0.14 |
| *Eugenia caryophyllata* Thunb. | 1.71±0.06** | *Vitis vinifera* L | 1.26±0.05 |
| *Vaccinium angustifolium* Ait | 1.22±0.05 |  |  |

A549 cells were seeded in 96-well plate for 24h, then cotransfected with pMC-Beclin1 and pMN-Bcl2 plasmids, after 6h, in the blank group (BG, untreated), A549 cells were not infected with IAV; in the negative control (NC, virus only) group, A549 cells were infected, but not treated with any drugs; in the positive control (PC, ribavirin) and test drug groups, A549 cells were infected, and treated with ribavirin (25 μg/ml) and test drugs at the maximum no-cytotoxicity concentrations, respectively. MOI = 2.0, the incubation times were 8h. The FI was determined at 610nm after excitation at 587nm using a microplate reader (Tecan infinite M1000). All numbers were expressed as fold change relative to the negative control (NC, virus only). Data shown were mean ± SD from three independent experiments performed in triplicate.* *P* < 0.05, ** *P* < 0.01 *vs.* the negative control (NC, virus only).
